# Taxifolin impairs colonization of *Clostridium perfringens* in association with iron limitation-related physiological stress

**DOI:** 10.1128/spectrum.01301-26

**Published:** 2026-08-21

**Authors:** Harum Fadhilatunnur, Weichen Gong, Haruna Sato, Eiji Hishinuma, Naomi Matsukawa, Fu Namai, Julio Villena, Hui-Yu Huang, Hiroaki Komatsu, Keita Nishiyama, Haruki Kitazawa

**Affiliations:** 1 Laboratory of Animal Food Function, Graduate School of Agricultural Science, Tohoku University 13101, Sendai, Japan; 2 Division of Food Science and Technology, Faculty of Engineering and Technology, IPB University 64759, Bogor, Indonesia; 3 International Education and Research Centre for Food and Agricultural Immunology (CFAI), Graduate School of Agricultural Science, Tohoku University 13101, Sendai, Japan; 4 Advanced Research Center for Innovations in Next-Generation Medicine, Tohoku University 13101, Sendai, Japan; 5 Tohoku Medical Megabank Organization, Tohoku University 13101, Sendai, Japan; 6 Faculty of Agriculture, Shinshu University, Minamiminowa, Japan; 7 Laboratory of Respiratory Immunology, Division of Animal Immunology and Omics, CFAI, Graduate School of Agricultural Science, Tohoku University 13101, Sendai, Japan; 8 Graduate Institute of Metabolism and Obesity Sciences College of Nutrition, Taipei Medical University 38032, Taipei, Taiwan; 9 SANOH Corporation Co., Ltd., Minato-ku, Tokyo, Japan; National University of Singapore, Singapore, Singapore

**Keywords:** taxifolin, *Clostridium perfringens*, colonization, iron limitation, intestinal pathogens

## Abstract

Flavonoids are plant-derived polyphenols with diverse biological activities; however, the mechanisms by which individual compounds inhibit intestinal pathogens remain unclear. In this study, we found that taxifolin (dihydroquercetin) showed pronounced inhibitory activity against *Clostridium perfringens*, a major gram-positive enteropathogen, whereas most other bacterial species tested were less affected. Notably, at concentrations below those causing complete growth inhibition, taxifolin significantly impaired mucin adhesion and biofilm formation, indicating suppression of colonization-associated phenotypes rather than bactericidal activity. In an antibiotic-pretreated mouse infection model, oral administration of taxifolin significantly reduced *C. perfringens* intestinal colonization during the early phase of infection. Mechanistically, proteomic analysis of *C. perfringens* revealed marked alterations in surface-associated and extracellular proteins, including stress response factors (MreB, LytR, and ClpB), and enzymes linked to iron- and redox-dependent metabolism, consistent with an iron-limiting stress response. Scanning electron microscopy further revealed pronounced cell elongation in taxifolin-treated cells, consistent with impaired cell division under stress conditions. Importantly, Fe^2+^ supplementation partially restored bacterial growth, normal morphology, mucin adhesion, and biofilm formation, whereas supplementation with other divalent ions failed to restore growth. Similar phenotypes were observed following treatment with the iron-specific chelator 2,2′-bipyridyl. Collectively, our findings support a working model in which iron limitation-associated physiological stress contributes to the inhibitory effects of taxifolin on *C. perfringens* growth and colonization-related phenotypes. This study provides insights into the interaction between dietary flavonoids and clostridial pathogens, highlighting the potential of taxifolin as a plant-derived compound for limiting *C. perfringens* intestinal colonization.

**IMPORTANCE:**

*Clostridium perfringens*, a clinically important pathogen in both humans and animals, causes various histotoxic and enteric diseases, including gas gangrene and foodborne or non-foodborne diarrhea. Its control remains challenging due to increasing antimicrobial resistance and the need to preserve beneficial gut microbiota. Here, we identified taxifolin, a naturally occurring flavonoid, as a potential inhibitor of *C. perfringens* that suppressed its growth and colonization-associated phenotypes while exerting comparatively limited effects on most other tested bacterial species, including commensal bacteria. Proteomic and physiological analyses revealed stress responses associated with iron metabolism, redox balance, and cell division, which were accompanied by reduced mucin adhesion, biofilm formation, and bacterial proliferation. Our findings suggest that taxifolin can reduce the colonization capacity of *C. perfringens* and support its potential use as a plant-derived approach for limiting *C. perfringens* intestinal colonization in humans and animals.

## INTRODUCTION

Flavonoids constitute a major class of dietary polyphenols abundant in fruits, vegetables, tea, and other plant-derived foods. They are traditionally recognized for antioxidant activities, including direct radical scavenging activity, modulation of cellular redox enzymes, metal chelation, and enhancement of endogenous antioxidant defenses (1, 2). Consistently, high flavonoid intake is associated with a reduced risk of oxidative stress-related pathologies, such as cardiovascular diseases, cancer, diabetes, and neurodegeneration (1–5). In addition to these host-centered effects, flavonoids exert microbiota-modulating effects. They selectively stimulate or inhibit distinct bacterial taxa, reshape microbial metabolic outputs, and undergo biotransformation by commensal microbes into secondary metabolites with altered bioactivity (6–9). Several flavonoids directly affect bacterial physiology by suppressing growth, modulating virulence-associated pathways, interfering with quorum sensing, or inhibiting biofilm formation in pathogenic species (10–13). Overall, flavonoids serve as diet-derived bioactive compounds that influence both microbial communities and microbe–host interactions.

Taxifolin (dihydroquercetin) is a naturally occurring dihydroflavonol in onions, grapes, citrus fruits, green tea, Siberian larch, and milk thistle (2, 14, 15). Previous studies have primarily focused on its antioxidant and cytoprotective effects. Taxifolin exhibits strong radical-scavenging activity and metal-chelating capacity (16), mitigates oxidative tissue damage by activating NF-E2-related factor 2-dependent signaling, and suppresses lipid peroxidation (17, 18). It also confers protection in various metabolic stress and inflammation models (19, 20). However, its effects on bacterial physiology and antimicrobial mechanisms remain unclear. Although moderate inhibitory effects against several gram-positive and gram-negative pathogens have been reported (15, 21), the underlying basis for bacterial susceptibility to taxifolin remains ambiguous. Taxifolin also reduces biofilm formation and attenuates *Staphylococcus aureus* virulence by inhibiting sortase A (22). However, its effects on gut-associated pathogens and commensal bacteria, particularly the determinants of species-specific sensitivity, remain unknown.

In this study, we evaluated the antimicrobial spectrum of taxifolin against representative gut commensal and pathogenic bacteria. We found that taxifolin showed the strongest inhibitory activity against *Clostridium perfringens* among the gut-associated bacteria tested. It suppressed *C. perfringens* growth, mucin adhesion, and biofilm formation and induced distinct physiological responses associated with growth-limiting stress. Taken together, our findings revealed a previously unrecognized susceptibility of *C. perfringens* to taxifolin and provide mechanistic insight into the inhibitory effects of this dietary flavonoid.

## MATERIALS AND METHODS

### Microorganisms and culture conditions

All bacterial strains, except *Clostridioides difficile* ATCC 9689^T^ (American Type Culture Collection, Manassas, VA, USA) and *C. sporogenes* NCTC 12935 (National Collection of Type Cultures, Salisbury, UK), were obtained from the Japan Collection of Microorganisms (JCM) or derived from laboratory stocks maintained at the Laboratory of Animal Food Function, Tohoku University (Sendai, Japan). Enterotoxigenic *Escherichia coli* 1263, enterohemorrhagic *E. coli* ATCC 43895, *Klebsiella pneumoniae* JCM 1662^T^, *Morganella morganii* JCM 1672^T^, and *Salmonella* enterica serovar Typhimurium SL1344 were aerobically cultured in the Luria–Bertani broth (Nacalai Tesque, Inc., Kyoto, Japan). *Bacillus cereus* JCM 2152^T^ and *S. aureus* JCM 16,555 were aerobically cultured in the modified Gifu anaerobic medium (mGAM; Shimadzu Diagnostics, Tokyo, Japan). *C. perfringens* JCM 1290^T^, JCM 3816, JCM 3817, JCM 5242, *C. difficil*e ATCC 9689^T^, *C. butyricum* JCM 1391^T^, and *C. sporogenes* NCTC 12935 were cultured in mGAM or GAM (Shimadzu Diagnostics) under strictly anaerobic conditions. *C. difficile* ATCC 9689^T^ was streaked onto Brucella HK agar medium RS (Kyokuto Pharmaceutical Industrial Co., Tokyo, Japan) and cultured in GAM broth for subsequent experiments. *Lactiplantibacillus plantarum* P27, *Ligilactobacillus salivarius* S22, *Bacteroides dorei* JCM 13471^T^, *Bacteroides thetaiotaomicron* JCM 5827^T^, *Bifidobacterium adolescentis* JCM 1275^T^, *B. longum* JCM 1217^T^, and *Parabacteroides johnsonii* JCM 13406^T^ were anaerobically cultured in the de Man–Rogosa–Sharpe broth (Difco; Becton, Dickinson and Company, Sparks, MA, USA) or mGAM, as appropriate. All anaerobic cultures were maintained in an anaerobic chamber (Coy Laboratory Products, Inc., Grass Lake, MI, USA) at 37°C.

### Taxifolin

Taxifolin (food grade; SANOH Corporation Co., Ltd., Tokyo, Japan) stock solution was prepared in dimethyl sulfoxide (FUJIFILM Wako Pure Chemical Corporation, Osaka, Japan) at 50 mg/mL and stored at –30°C until use. Working concentrations were obtained by diluting the stock in the growth medium, as required. The same volume of dimethyl sulfoxide was added to the control samples to equalize solvent concentration.

### Bacterial growth assay

For each bacterium, overnight cultures from glycerol stock were subcultured into their respective broth media and incubated at 37°C for 24 h. Then, 2 µL (2%) of each culture was inoculated into 100 µL of medium containing different concentrations of taxifolin (0.062, 0.125, 0.25, 0.5, and 1 mg/mL) in 96-well plates (Violamo; AS ONE, Osaka, Japan), with medium without taxifolin serving as the control. Bacterial growth was monitored every 30 min by measuring the optical density at 600 nm (OD_600_) using a microplate reader (Cerillo, Charlottesville, VA, USA) at 37°C. The growth of anaerobic bacteria was measured in an anaerobic chamber (Coy Laboratory Products, Inc.). Based on OD_600_ measurements, the minimal inhibitory concentration causing ≥70% growth inhibition (MIC_70_) was determined at the time point when the untreated control reached its maximal OD_600_.

### Time-kill assay

Based on the initial growth assay, subsequent experiments were performed using *C. perfringens* JCM 1290^T^. To determine whether the effect of taxifolin was bacteriostatic or bactericidal, a time-kill assay was conducted. *C. perfringens* was cultured anaerobically at 37°C in mGAM containing an inhibitory concentration of taxifolin. At designated time points (0, 4, 8, and 24 h), bacterial growth or inhibition was monitored by measuring the OD_660_ values (Mini Photo 518R; TAITEC, Saitama, Japan), and viable cell counts were determined by the plate count method using mGAM agar.

### Scanning electron microscopy (SEM) imaging

*C. perfringens* JCM 1290^T^ cells in the late exponential phase were fixed overnight in freshly prepared 2.5% glutaraldehyde (FUJIFILM Wako Pure Chemical Corporation) in phosphate-buffered saline (PBS) (Life Technologies Corporation, Grand Island, NY, USA) at 4°C. After fixation, the cells were collected via centrifugation at 4,000 × *g* for 5 min at 4°C, washed with PBS, and transferred to 0.1 M cacodylate buffer (pH 7.4; FUJIFILM Wako Pure Chemical Corporation). The samples were treated with 1% tannic acid (FUJIFILM Wako Pure Chemical Corporation) to enhance conductivity and fixed with 1% osmium tetroxide (Nissin EM Co., Ltd., Tokyo, Japan) in the same buffer at 4°C for 2 h. Following dehydration through a graded ethanol series to absolute ethanol, the specimens were infiltrated with tert-butyl alcohol, frozen at –10°C, and dried using a vacuum freeze-dryer (VFD-21S; Vacuum Device, Ibaraki, Japan). The dried samples were mounted on aluminum stubs, coated with a thin osmium layer using a conductive coater (Neoc-ST; Meiwafosis, Tokyo, Japan), and observed under a scanning electron microscope (SU6600; Hitachi High-Tech, Tokyo, Japan) at 5 kV.

### Adhesion assay

Bacterial adhesion to intestinal mucin was evaluated as previously described by Nishiyama and Mukai (23). Each well of diagnostic glass was coated with 40 µL of mucin solution (1 mg/mL) and incubated at 4°C for 12 h. After washing with washing buffer (0.1% [wt/vol] bovine serum albumin [BSA; Sigma-Aldrich, St. Louis, MO, USA] in PBS) and drying, the wells were blocked with 40 µL of blocking buffer (5% [wt/vol] BSA in PBS) at room temperature (25°C) for 2 h. After washing with washing buffer, 40 µL of overnight *C. perfringens* JCM 1290^T^ culture (adjusted to OD_600_ = 1) was added to each well and incubated for 2 h at 37°C. Non-adherent bacteria were removed by washing, and the remaining cells were stained with methylene blue (Sigma-Aldrich) and visualized under a light microscope (Olympus BX40; Olympus Optical Co., Ltd., Tokyo, Japan). Adhesion was quantified as the percentage of area covered by adherent bacteria, as determined using ImageJ software (National Institute of Health, Bethesda, MD, USA).

### Biofilm assay

*C. perfringens* JCM 1290^T^ was grown in taxifolin-containing mGAM in a 96-well collagen-coated plate (IWAKI; AGC Techno Glass Co., Shizuoka, Japan) under anaerobic conditions at 37°C for 72 h. Wells containing the same medium without bacteria served as blanks. After removing the medium, biofilms were gently washed thrice with water. Then, the biofilms were stained with 5% crystal violet (Muto Pure Chemicals Co., Ltd., Tokyo, Japan) at room temperature (25°C) for 15 min and washed again with water (thrice). The bound stain was solubilized with 90% ethanol (FUJIFILM Wako Pure Chemical Corporation), and absorbance was measured at 560 nm using a microplate reader (PerkinElmer, Shelton, CT, USA). Biofilm formation was quantified after normalization to the blanks.

### *In vivo* experiment

Male C57BL/6 mice (10 weeks old) were purchased from CLEA Japan Inc. (Tokyo, Japan). Mice were maintained under specific pathogen-free conditions (22–24°C, 40–60% humidity, 12-h light/dark cycle) and provided *ad libitum* access to standard rodent chow (CL-2, 50 kGy irradiated; CLEA Japan, Inc.) and water. All efforts were made to minimize animal suffering.

To determine whether taxifolin reduces *C. perfringens* intestinal colonization *in vivo*, a mouse challenge model modified based on a previous method (24) was used. Mice (*n* = 12/group) were administered a mixed-antibiotic regimen consisting of vancomycin (45 mg/L; FUJIFILM Wako Pure Chemical Corporation), colistin (35 mg/L; FUJIFILM Wako Pure Chemical Corporation), gentamicin (35 mg/L; Tokyo Chemical Industry Co., Tokyo, Japan), and metronidazole (215 mg/L; FUJIFILM Wako Pure Chemical Corporation) in drinking water for 7 days. The mice received taxifolin (1 mg/mL) or water in their drinking water until the end of the experiment. Two days after completing antibiotic pretreatment, the mice were intraperitoneally administered clindamycin (0.016 mg/g body weight; Tokyo Chemical Industry Co.). The following day, the mice were orally challenged once with approximately 8 × 10^7^ colony-forming units (CFU) of *C. perfringens* JCM 1290ᵀ suspended in 200 μL of PBS. For bacterial preparation, *C. perfringens* was cultured from glycerol stock in mGAM broth under anaerobic conditions at 37°C. Overnight cultures (2%) were transferred to fresh medium and grown to mid-exponential phase. Bacterial cells were harvested via centrifugation at 10,000 × *g* for 5 min at 4°C and washed twice with PBS before infection.

Fecal *C. perfringens* levels were quantified on days 1, 3, 5, and 7 post-challenge by counting on CW agar plates (Vital Media CW agar; Kyokuto Pharmaceutical Industrial Co.), a selective medium for *C. perfringens* (Fig. 2D). Fecal samples were resuspended in 1 mL PBS, briefly centrifuged at 1,000 × *g* for 1 min to remove debris, and the supernatants were subjected to serial dilutions. Aliquots of each dilution were plated on CW agar and anaerobically incubated at 37°C for 48 h. White colonies were counted as *C. perfringens*. Colony identity was confirmed via polymerase chain reaction amplification, followed by Sanger sequencing of the *16S rRNA* gene.

### Extraction of bacterial proteins

To elucidate the mechanistic effects of taxifolin at the molecular level, cell surface-associated, secreted, and whole-cell lysate (WCL) proteins of *C. perfringens* were extracted, as outlined in Fig. 3A. *C. perfringens* JCM 1290^T^ was anaerobically cultured in mGAM at 37°C until the late exponential phase (5 h for the control; 6 and 7 h for 0.062 and 0.125 mg/mL taxifolin, respectively). The cultures were centrifuged at 6,000 × *g* for 5 min, after which the cell pellets were separated from the supernatant. The supernatants were further filtered through a 0.22 µm membrane (Toyo Roshi, Kaisha, Ltd., Tokyo, Japan). Both the cell pellets and clarified supernatants were stored at –30°C until protein extraction.

Cell surface-associated proteins were extracted by resuspending the cell pellets (from 1 mL of bacterial culture) in 200 µL of extraction buffer containing PBS, 30% sucrose, 0.05 mg lysozyme (Sigma-Aldrich), 25 U mutanolysin (Sigma-Aldrich), and a protease inhibitor cocktail (ATTO, Tokyo, Japan). The suspension was incubated at 37°C for 3 h and centrifuged at 16,000 × *g* for 10 min at 4°C. The supernatant, representing cell surface-associated proteins, was collected for further analysis.

Secreted proteins were concentrated using a standard trichloroacetic acid precipitation method. Briefly, 1,500 µL of bacterial supernatant was mixed with 300 µL of trichloroacetic acid (FUJIFILM Wako Pure Chemical Corporation) and incubated on ice for 1 h. The precipitated proteins were collected via centrifugation at 20,000 × *g* for 5 min at 4°C, washed twice with 400 µL of ice-cold acetone (Wako Pure Chemical Industries, Osaka, Japan), and re-centrifuged. Acetone was removed, and the pellet was air-dried. The resulting protein precipitates were dissolved in 100 µL of 0.05 M NaOH for sodium dodecyl sulfate (SDS)–polyacrylamide gel electrophoresis analysis or PBS for proteomic analysis.

WCL proteins were extracted by resuspending the pelleted cells (from 1 mL of bacterial culture) in 200 µL of extraction buffer containing PBS, 0.05 mg lysozyme, 25 U mutanolysin, and a protease inhibitor cocktail, followed by incubation at 37°C for 1 h. The cells were disrupted by bead-beating using 0.3 g of 0.1 mm zirconia-silica beads (Bio Spec Products, Bartleville, OK, USA) at 4,800 rpm for 30 s, repeated four times at 60-s intervals on ice. The supernatant, representing WCL proteins, was collected after centrifugation at 16,000 × *g* for 5 min at 4°C. All proteins were stored at –30°C until use. Protein concentrations were determined using the Pierce BCA Protein Assay Kit (Thermo Fisher Scientific, Waltham, MA, USA) and standardized to 1 mg/mL.

### SDS–PAGE

Extracted proteins were mixed 1:1 (vol/vol) with 2 × sample buffer solution (2ME+) (FUJIFILM Wako Pure Chemical Corporation) and denatured at 95°C for 5 min. Equal amounts of protein (approximately 10–20 µg/lane) were loaded onto 10% Mini-PROTEAN TGX Precast Protein Gels (Bio-Rad Laboratories, Hercules, CA, USA) and separated via electrophoresis at 130 V, 0.04 A, and 6 W. After electrophoresis, the gels were stained with a silver staining kit (ATTO) according to the manufacturer’s instructions.

### Proteomics

Cell wall-associated, secreted, and WCL proteins in the control and taxifolin-treated groups were subjected to proteomic analyses. Protein concentrations were adjusted to 500 µg/mL with PBS, followed by purification via chloroform/methanol precipitation. The samples were processed using the iST Sample Preparation Kit (PreOmics, Planegg, Germany), according to the manufacturer’s instructions. Briefly, proteins were dissolved in lysis buffer and incubated at 95°C for 10 min. The reduced and alkylated protein samples were digested with trypsin/LysC at 37°C for 3 h. The resulting peptide mixtures were washed with wash solutions, eluted with elution buffer, and dried before liquid chromatography–tandem mass spectrometry analysis. Peptides were analyzed using the Orbitrap Fusion mass spectrometer (Thermo Fisher Scientific) coupled with a nanoflow high-performance liquid chromatography system (EASY-nLC 1000; Thermo Fisher Scientific), as previously described (25, 26). Raw mass spectrometry data were processed using Proteome Discoverer version 3.0 (Thermo Fisher Scientific) with the Sequest HT search algorithm against the UniProt database (https://www.uniprot.org/) for *C. perfringens* (taxonomy ID: 1502), downloaded on July 25, 2025. Proteomic data analysis was performed using GraphPad Prism 8 (GraphPad Software, Inc., San Diego, CA, USA) to generate volcano plots. Principal component analysis (PCA) was conducted using the SIMCA software version 13.0.3 (Sartorius, San Francisco, CA, USA). Quantified protein abundances in the taxifolin-treated group were compared to those in the control group. Statistical significance was defined as a fold change (treated/control) <0.5 or >2 (log_₂_ fold change < −1 or >1) with a *P* value < 0.05. Additionally, the proteomic data were subjected to Benjamini–Hochberg false discovery rate (FDR) correction. Volcano plots are based on nominal *P* values.

### Metal ion and chelator experiments

Metal ion and chelator assays were performed as previously described (27–29), with minor modifications. The concentrations of reagents used in this study were determined according to the conditions reported in the literature. A 10 mM Fe^2+^ stock solution was prepared by dissolving FeSO_4_·7H_2_O (FUJIFILM Wako Pure Chemical Corporation) in Milli-Q water (Merck, Darmstadt, Germany) followed by the addition of 37% HCl (FUJIFILM Wako Pure Chemical Corporation) to stabilize the solution. Additionally, 300 µM 2,2′-bipyridyl (BP; Tokyo Chemical Industry Co.) was prepared by dissolving it in Milli-Q water. Both solutions were filter-sterilized using a 0.22 µm membrane filter and stored at 4°C protected from light.

The growth of *C. perfringens* JCM 1290^T^ or *C. difficile* was assessed in the presence of taxifolin at their inhibitory concentrations (0.5 and 1 mg/mL, respectively), Fe^2+^ (5 and 10 µg/mL), their combination, or BP (300 or 500 µM). A 2% inoculum (2 µL) of an overnight culture was added to 100 µL of mGAM broth containing the respective treatments in 96-well plates. Bacterial growth was monitored every 30 min by measuring OD_600_ using a microplate reader (Cerillo) at 37°C under anaerobic conditions in an anaerobic chamber (Coy Laboratory Products, Inc.).

To confirm the essential role of iron in the growth of *C. perfringens*, the bacterium was cultured under TSB-based iron-depleted and -repleted conditions (28). *C. perfringens* was initially grown in fluid thioglycolate broth (Becton, Dickinson and Company) for 6 h, after which a 2% inoculum was transferred into 100 µL of trypticase soy broth (TSB; control), TSB supplemented with 300 µM BP (iron-depleted), or TSB containing 300 µM BP and 10 µg/mL FeSO_4_·7H₂O (iron-repleted) in 96-well plates. Simultaneously, taxifolin (0.5 mg/mL) was added to evaluate its potential iron-chelating activity. Bacterial growth was monitored every 30 min by measuring OD_600_ at 37°C under anaerobic conditions.

The cell morphology of *C. perfringens* cultured under similar growth conditions was observed using a light microscope (Olympus BX40; Olympus Optical Co., Ltd.). *C. perfringens* was cultured anaerobically at 37°C for 24 h in mGAM containing BP (200 and 300 µM) or taxifolin (0.125 and 0.25 mg/mL), with or without Fe^2+^ (5 µg/mL) supplementation. Bacterial cells were stained with crystal violet and observed at 1,000× magnification.

To evaluate the effect of iron on colonization-associated phenotypes, mucin adhesion and biofilm formation of *C. perfringens* JCM 1290^T^ were assessed in iron-limited and -repleted conditions. *C. perfringens* was cultured in mGAM containing different concentrations of BP (100–300 µM) or taxifolin (0.062–0.25 mg/mL) to establish iron-limited conditions. Fe^2+^ (5 µg/mL) was supplemented into each taxifolin-containing medium to restore iron availability. Mucin adhesion and biofilm formation were quantified as described in the respective adhesion and biofilm assay sections.

Free iron concentration was measured using ferrozine (Dojindo Laboratories Co., Ltd., Kumamoto, Japan) as previously described (30), with some modifications. A ferrozine stock solution was prepared by diluting ferrozine in 10 mM acetate buffer. The solution was stored at 4°C in an amber tube until future use. Then, standard Fe solutions were prepared from the FeSO_4_·7H_2_O stock solution to construct a calibration curve. Samples containing 10 µg/mL Fe^2+^, 300 µM BP, 0.5 mg/mL taxifolin, or their combination were prepared. Ascorbic acid was added to each solution to a final concentration of 5 mM to prevent Fe^2+^ oxidation. An aliquot (10 µL) of each solution was transferred to a 96-well plate with 90 µL of 2 mM ferrozine solution. Then, absorbance was measured at 560 nm. Because BP and taxifolin form color upon reacting with Fe^2+^, all absorbances were normalized using blanks containing each solution without ferrozine.

To determine whether divalent ions other than iron contribute to the inhibitory action of taxifolin, similar growth rescue experiments were performed with Mg^2+^, Ca^2+^, Mn^2+^, and Zn^2+^. Stock solutions of each mineral were prepared from MgCl_2_ (FUJIFILM Wako Pure Chemical Corporation), CaCl_2_ (FUJIFILM Wako Pure Chemical Corporation), MnCl_2_·4H_2_O (FUJIFILM Wako Pure Chemical Corporation), and ZnCl_2_ (FUJIFILM Wako Pure Chemical Corporation) at the concentrations of 1 mg/mL in Milli-Q water. The solutions were filter-sterilized with a 0.22 µm filter and supplemented into the medium at final concentrations of 0, 5, 10, 15, and 20 µg/mL in the presence of 0.5 mg/mL taxifolin.

### Cytotoxic effects of taxifolin on intestinal epithelial cells

The cytotoxicity of taxifolin was determined using Cell Counting Kit-8 assay (Dojindo Laboratories Co., Ltd.). Porcine intestinal epithelial (INV-1-3) cells were cloned from the small intestine of pigs as previously described (31). The cells were seeded at a density of 6.8 × 10^5^ cells/mL in a 96-well collagen-coated plate. The plate was incubated at 37°C for 72 h in an atmosphere containing 5% CO_2_. The culture medium was exchanged with fresh medium containing various concentrations of taxifolin (0.062, 0.125, and 0.25 mg/mL) and incubated for an additional 48 h. Then, the Cell Counting Kit-8 solution (10 µL) was added to each well, and the plate was incubated for 1 h under the same conditions. Finally, absorbance was measured at 450 nm.

### Statistical analyses

All statistical analyses were conducted using GraphPad Prism (GraphPad Software, Inc.). Data are represented as the mean ± standard deviation, as indicated in the figure legends. For comparisons between two groups, a two-tailed Student’s *t*-test was used when the data followed a normal distribution. For comparisons among multiple groups, one-way analysis of variance followed by appropriate post-hoc tests (Dunnett’s or Tukey’s multiple comparisons test) was used, as specified in each figure legend. For *in vivo* fecal bacterial counts, which did not exhibit a normal distribution, the non-parametric Mann–Whitney *U* test was used. Proteomic data were analyzed using a two-tailed Student’s *t*-test, and proteins were considered significantly altered at *P* < 0.05 with an absolute log_2_ fold change >1. The number of biological replicates (*n*) for each experiment is indicated in the figure legends. Statistical significance was set at *P* < 0.05.

## RESULTS

### Effect of taxifolin on the growth of pathogenic and commensal bacteria

**Fig 1:**
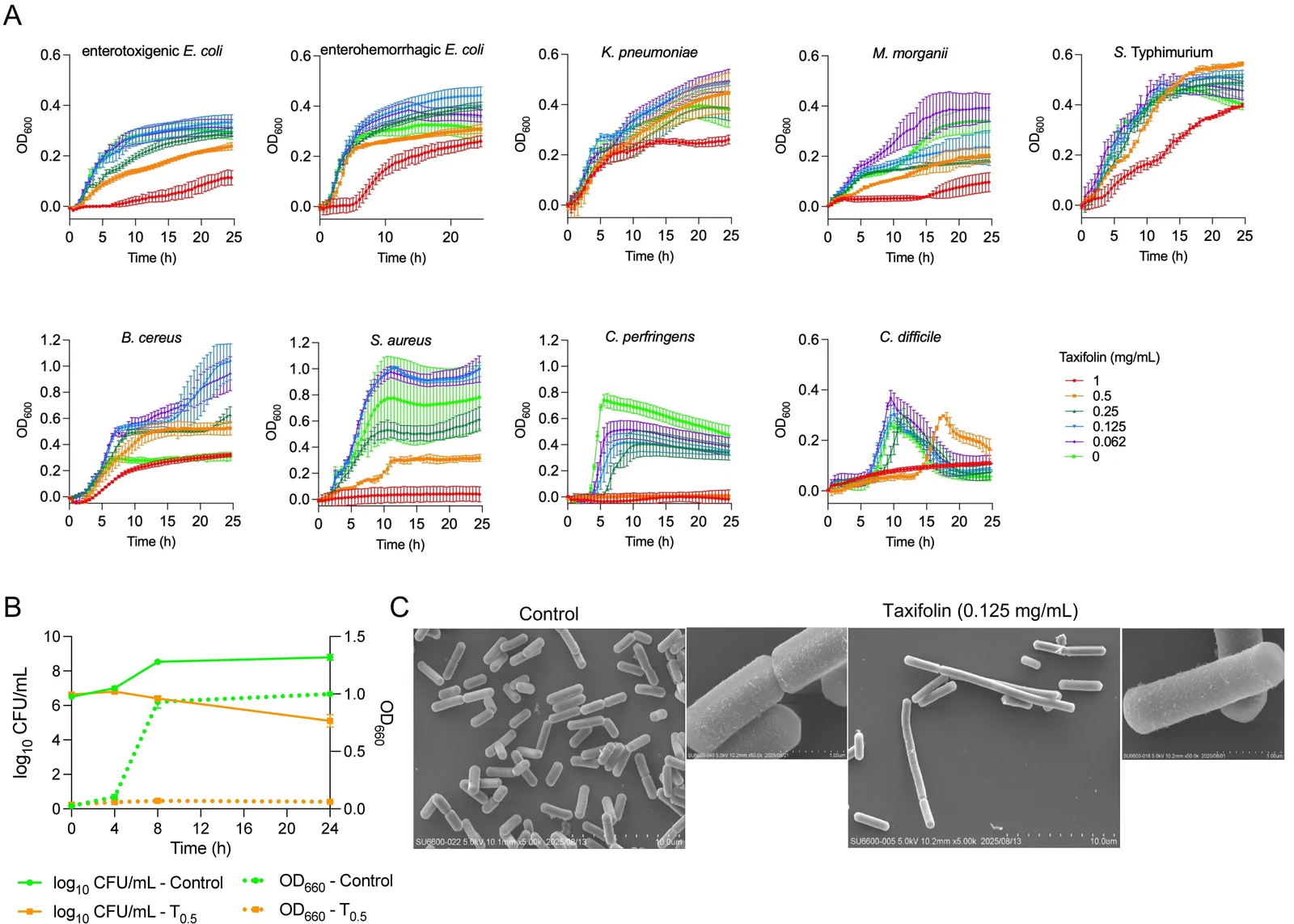
Effects of taxifolin on the growth and cell morphology of pathogenic bacteria. (**A**) Growth kinetics of representative gram-negative and gram-positive pathogens cultured in the presence of increasing concentrations of taxifolin (0–1 mg/mL). Optical density at 600 nm (OD_600_) was recorded every 30 min to monitor bacterial proliferation. Data are represented as the mean ± standard deviation (SD) of three independent biological replicates. The corresponding MIC_70_ values, defined as the concentrations yielding 70% inhibition at the time when the untreated control reached maximal OD_600_, are presented in Fig. S1. (**B**) Colony counts (log_10_ CFU/mL) and optical density (OD_660_) of *Clostridium perfringens* cultured at several time intervals with 0 (control) and 0.5 mg/mL taxifolin (T_0.5_). Data represent the mean ± SD of three independent experiments, with each dot indicating an individual replicate. (**C**) Scanning electron micrographs of *C. perfringens* cells cultured with 0 (control) and 0.125 mg/mL taxifolin. Untreated cells exhibited short rod-shaped morphology with smooth surfaces, whereas taxifolin-treated cells exhibited elongated morphology with irregular surface textures and occasional incomplete septation. Scale bars, 1 and 10 µm. Representative images from 20 independent images are shown.

The antibacterial activity of taxifolin was assessed against representative gram-negative and -positive pathogens. Bacterial growth in the presence of increasing concentrations of taxifolin (0-1 mg/mL) was monitored by measuring OD_600_ using a broth microdilution assay. As shown in Fig. 1A, taxifolin exerted negligible effects on gram-negative bacteria, causing only a slight growth delay at 1 mg/mL, particularly in enterotoxigenic *E. coli*, enterohemorrhagic *E. coli*, and *M. morganii*. In contrast, gram-positive bacteria were considerably more susceptible to taxifolin. Notably, *C. perfringens* exhibited the highest sensitivity, showing complete growth inhibition at taxifolin concentrations ≥0.5 mg/mL, whereas *C. difficile*, *S. aureus*, and *B. cereus* exhibited noticeable growth retardation at 0.5 mg/mL (Fig. 1A). MIC_70_ was determined when the untreated control reached maximal OD_600_. *C. perfringens* showed the lowest MIC_70_ value (0.125 mg/mL; Fig. S1), confirming its pronounced sensitivity to taxifolin.

A panel of intestinal isolates was tested under the same assay conditions to evaluate the effects of taxifolin on representative gut commensal species. As shown in Fig. S2, most commensal bacteria, including *L. salivarius*, *L. plantarum*, *B. adolescentis*, *B. longum*, *B. dorei*, *P. johnsonii*, and *B. thetaiotaomicron*, exhibited growth kinetics comparable to those of untreated controls in the presence of up to 1 mg/mL taxifolin. A slight delay in the exponential phase was observed in some strains at the highest concentration; however, complete growth inhibition was not observed. In contrast, *C. butyricum* and *C. sporogenes* were more sensitive, showing reduced growth at taxifolin concentrations ≥0.5 mg/mL. Similar growth suppression patterns were observed in other *C. perfringens* strains, including JCM 5242, JCM 3816, and JCM 3817, although the magnitude of inhibition varied among strains (Fig. 1; Fig. S1 and S3). Based on its greater susceptibility to taxifolin than other bacterial species and *C. perfringens* strains tested, *C. perfringens* JCM 1290^T^ was selected for subsequent mechanistic analyses.

Next, we investigated the effects of taxifolin on *C. perfringens*. Bacterial viability was evaluated using a time-kill assay at the inhibitory concentration of 0.5 mg/mL. As shown in Fig. 1B, treatment with taxifolin completely suppressed the increase in OD_660_; however, substantial numbers of viable bacteria remained throughout the incubation period. Bacterial counts remained relatively constant during the first 8 h of exposure to taxifolin and subsequently decreased by less than 3 log_10_ CFU/mL relative to the initial inoculum after 24 h. In contrast, the untreated control exhibited marked increases in both viable cell counts and OD_660_. Because a bactericidal effect is generally defined as a reduction of ≥3 log_10_ CFU/mL within 24 h, these results indicate that taxifolin exerts a predominantly bacteriostatic rather than bactericidal effect against *C. perfringens*.

To visualize the morphological alterations associated with this bacteriostatic effect, *C. perfringens* JCM 1290ᵀ cells treated with the MIC_70_ concentration (0.125 mg/mL) were examined via SEM. Untreated cells exhibited the characteristic short rod-shaped morphology with smooth intact surfaces, whereas taxifolin-treated cells were noticeably elongated (Fig. 1C). Quantitative measurements confirmed a significant increase in cell length (*P* < 0.0001; Fig. S4), indicating disrupted septum formation and impaired cell division. Together, these morphological changes reflect growth inhibition rather than cell lysis and are consistent with the bacteriostatic effect of taxifolin.

### Taxifolin attenuates *C. perfringens* colonization *in vitro* and *in vivo*

**Fig 2:**
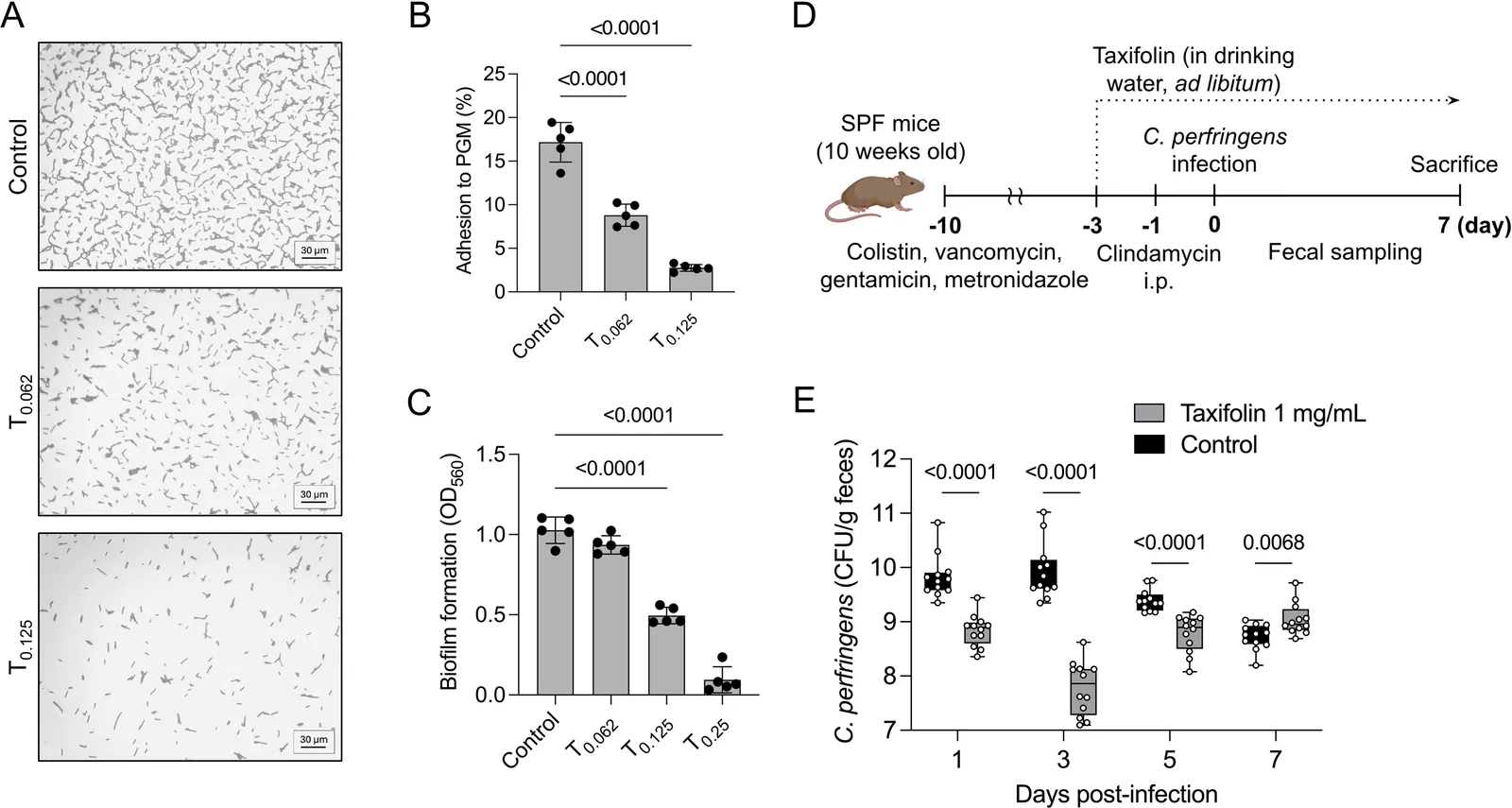
Effects of taxifolin on *C. perfringens* adhesion, biofilm formation, and intestinal colonization. (**A**) Representative microscopic images showing bacterial adhesion to porcine gastric mucin after treatment with the indicated concentrations of taxifolin (0.062 and 0.125 mg/mL; 400× magnification). (**B**) Quantification of bacterial adhesion shown in panel **A** expressed as the percentage of mucin surface area covered by adherent bacterial cells. Each dot represents the value quantified from an individual microscopic image acquired from a distinct well. Bars represent the mean ± SD. (**C**) *C. perfringens* biofilm formation on collagen type I in the presence of increasing concentrations of taxifolin. Bars represent the mean ± SD of five independent experiments, with each dot indicating an individual replicate. (**B and C**) Statistical significance was determined via one-way analysis of variance (ANOVA), followed by Dunnett’s post-hoc test compared to the control group. (**D**) Schematic overview of the mouse challenge experiment. (**E**) Fecal *C. perfringens* counts (colony-forming units [CFU]/g of feces) on days 1, 3, 5, and 7 post-challenge. Data shown represent pooled results from two independent experiments, with each dot corresponding to an individual mouse. Box plots show the median with interquartile range (IQR), with whiskers indicating the minimum and maximum values (*n* = 12/group). Statistical significance was determined via Mann–Whitney *U* test. Abbreviations: C, control; T_0.062_, 0.062 mg/mL taxifolin; T_0.125_, 0.125 mg/mL taxifolin; T_0.25_, 0.25 mg/mL taxifolin.

Adhesion to intestinal mucus and subsequent biofilm formation are important steps in *C. perfringens* colonization and persistence in the intestinal environment (32, 33). To determine whether taxifolin interferes with colonization-related processes at concentrations below those causing complete growth inhibition (subinhibitory concentrations), we examined its effects at concentrations up to 0.25 mg/mL. Microscopic observations revealed a marked reduction in bacterial adhesion to porcine gastric mucin (PGM), beginning at 0.062 mg/mL taxifolin, with adhesion decreasing progressively in a dose-dependent manner (Fig. 2A). Consistently, quantitative analysis confirmed a significant decrease in the proportion of mucin surfaces covered by adherent cells (Fig. 2B). Taxifolin also markedly reduced biofilm formation by *C. perfringens* (Fig. 2C). Significant reductions in biofilm biomass were observed from 0.125 mg/mL onward, with further decreases at higher concentrations. Taken together, these results suggest that taxifolin significantly impairs *C. perfringens* mucin adhesion and biofilm formation at subinhibitory concentrations in a dose-dependent manner, suggesting that these effects were not solely attributable to bacterial killing.

Next, we assessed the effects of taxifolin on *C. perfringens* infection dynamics *in vivo*. Following antibiotic pretreatment, the mice received either taxifolin (1 mg/mL) or water *ad libitum* throughout the experiment. Taxifolin was supplied in drinking water, which was replaced with a freshly prepared solution every 2–3 days. The mice were challenged with *C. perfringens* via oral gavage on day 10 after the initial antibiotic pretreatment. Fecal plate counts were measured on days 1, 3, 5, and 7 post-infection (Fig. 2D). Notably, mice treated with 1 mg/mL taxifolin exhibited significantly lower *C. perfringens* counts in fecal samples than control mice (Fig. 2E). This difference was most pronounced on days 1 and 3 post-infection and gradually diminished over time, with bacterial counts becoming more comparable between the groups by day 7. These findings likely reflect impaired early colonization rather than sustained bactericidal effects, consistent with the observed inhibition of mucin adhesion and biofilm formation. Throughout the experimental period, both the taxifolin-treated and control groups maintained stable body weights, with no significant differences observed between the groups (*P* > 0.05), indicating that taxifolin administration did not exert overt adverse effects on the mice (Fig. S5).

### Proteomic analysis of taxifolin-exposed *C. perfringens*

**Fig 3:**
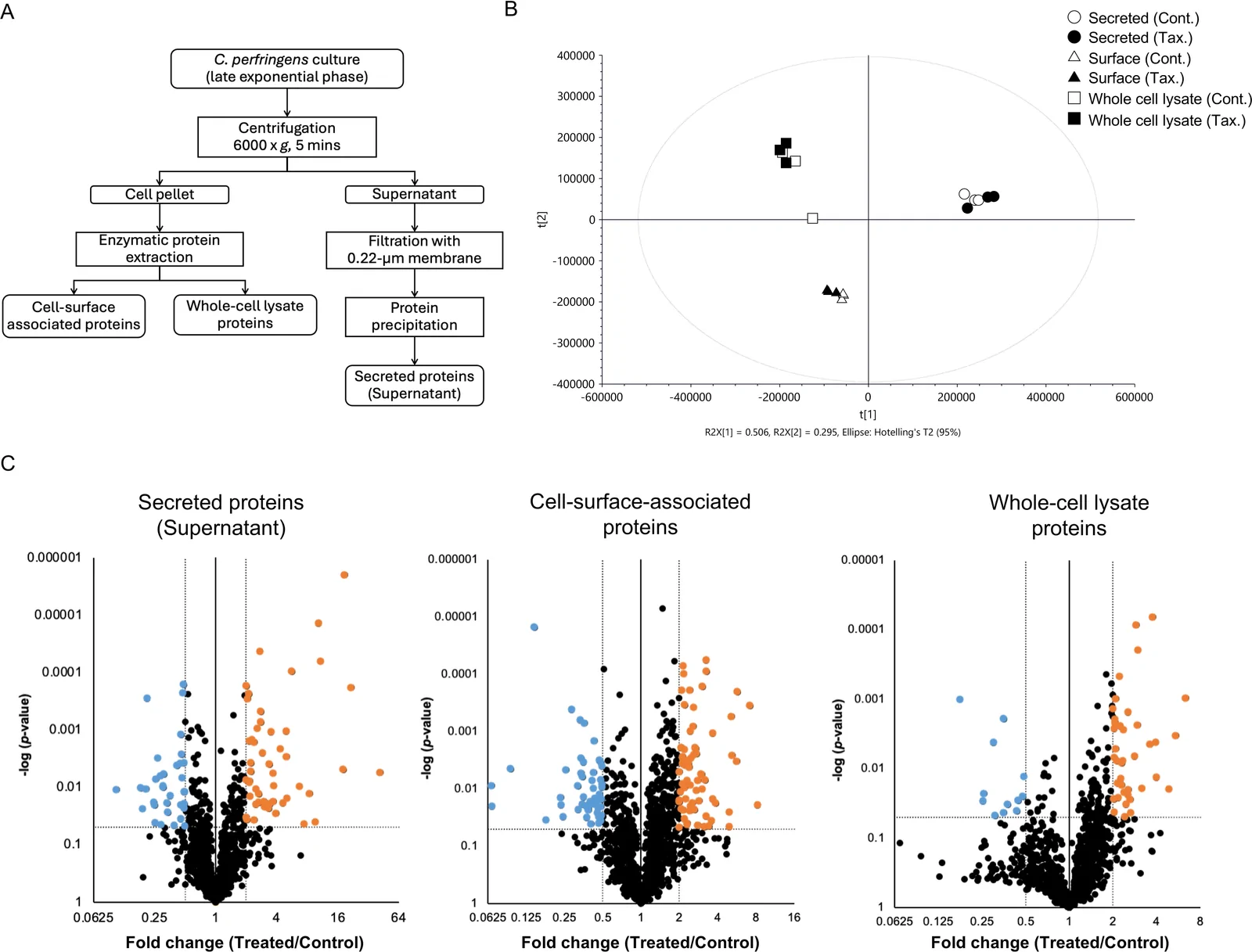
Global proteomic profiling of *C. perfringens* exposed to subinhibitory concentrations of taxifolin. (**A**) Schematic overview of the fractionation procedure used to obtain whole-cell lysate (WCL), cell surface-associated, and secreted proteins. (**B**) Principal component analysis (PCA) illustrating the global separation between taxifolin-treated (0.125 mg/mL) and untreated control groups across the three analyzed fractions: WCL, surface-associated, and secreted proteins. (**C**) Volcano plot showing the significantly altered proteins (nominal *P* < 0.05; log_2_ fold change < –1 or >1) identified via liquid chromatography–tandem mass spectrometry (LC–MS/MS)-based proteomic analysis. Proteins with increased abundance (orange) included stress-related and cell wall-associated factors, such as RapZ, LytR, and ClpB, whereas proteins with decreased abundance included enzymes involved in carbohydrate and nucleotide metabolism. Each point represents a quantified protein, and data reflect the mean of three independent biological replicates per group. All differentially expressed proteins are listed in Tables 1 to 3 and Tables S1 to S3.

We further investigated the molecular mechanisms underlying the antibacterial activity of taxifolin by performing comparative proteomic analyses of the whole-cell lysate (WCL), surface-associated, and secreted proteins of *C. perfringens* treated with 0.125 mg/mL taxifolin (Fig. 3A). The proteins from each fraction were separated via SDS–PAGE and visualized via silver staining (Fig. S6A), which revealed distinct banding patterns among the three fractions, reflecting their different subcellular localizations. Notably, taxifolin treatment induced visible alterations primarily in the surface-associated fraction, whereas only minor changes were observed in the WCL and secreted protein fractions (Fig. S6A). Global proteomic profiling confirmed successful fractionation, as PCA revealed a clear separation of the WCL, surface-associated, and secreted proteomes, regardless of treatment (Fig. 3B). PCA comparing the control and taxifolin-treated samples within each fraction further revealed distinct clustering between the two groups, with the largest variance observed in the surface-associated and extracellular proteomes (Fig. S6B). These results suggest that subinhibitory taxifolin exposure induces broad alterations in the surface-associated and secreted proteins of *C. perfringens*.

**TABLE 1:** Top 20 significantly abundant secreted proteins^*a*^ ranked by *P* value

Accession	Description	log_2_FC^*b*^	*P value* ^*c*^	Note
A0A133N153	RNase adapter RapZ	4.2341	0.000002	Up
A0A127EI13	3-Oxoacyl-[acyl-carrier-protein] reductase	3.3772	0.000014	Up
Q0SR38	Cell shape-determining protein MreB	1.4586	0.000043	Up
A0A0H2YSC8	Cell wall surface anchor family protein	3.4470	0.000064	Up
B1V7P4	Trigger factor	2.5014	0.000096	Up
Q8XKP4	Fructose-bisphosphate aldolase	−1.0448	0.000164	Down
A0A0N9KRK1	tRNA N6-adenosine threonylcarbamoyltransferase	1.0162	0.000172	Up
B1BQT4	Inosine-5′-monophosphate dehydrogenase	4.4509	0.000184	Up
A0AAV3BV35	Bis(5′-nucleosyl)-tetraphosphatase (symmetrical)	−1.0688	0.000228	Down
A0A0H2YT73	RNA-binding protein	1.0997	0.000236	Up
A0A133N789	4-Diphosphocytidyl-2-C-methyl-D-erythritol kinase	−2.2363	0.000283	Down
A0AAP6IVU1	LytR family transcriptional regulator	1.0564	0.000290	Up
A0A0H2YS43	von Willebrand factor type A/Cna B-type domain protein	1.4877	0.000484	Up
A0AAV3BQ85	DHH subfamily 1 protein	1.4888	0.000741	Up
A0A0H2YPM3	Chaperone protein ClpB	1.3628	0.000951	Up
A0A133NE19	Iron–sulfur cluster carrier protein	2.3208	0.001060	Up
A0AAN5SF70	L-lactate dehydrogenase	1.8243	0.001097	Up
A0A133N9F3	Small ribosomal subunit protein uS8	−1.1248	0.001219	Down
A0AAV3FBY1	AsnC family transcriptional regulator (fragment)	1.1978	0.001500	Up
A0A2X2Y3T7	Extracellular solute-binding protein	1.1103	0.001590	Up

^*a*^ Statistically significant proteins were characterized by *P* < 0.05 and log₂ FC < −1 or >1.

^*b*^ FC, fold change (treated/control group).

^*c*^ Statistical analysis was performed with two-tailed Student’s *t*-test from three independent experiments.

**TABLE 2:** Top 20 significantly abundant surface-associated proteins^*a*^ ranked by *P* value

Accession	Description	log_2_FC^*b*^	*P* value^*c*^	Note
A0A0H2YPA7	Single-stranded DNA-binding protein	−2.7776	0.000015	Down
A0A127EEU5	Glycosyl transferase family 1	1.6998	0.000057	Up
A0AAV3F981	Small ribosomal subunit protein uS12	1.0985	0.000071	Up
A0A0H2YSH2	ATP-cone domain-containing protein	1.7086	0.000089	Up
A0A133MWC8	Putative competence-damage inducible protein	1.1133	0.000098	Up
A0A127EK02	Uridine kinase	1.6045	0.000164	Up
A0A127EMB2	Lactate utilization protein	1.2775	0.000189	Up
A0AAN5N2F9	Uridylate kinase	1.0668	0.00019	Up
A0A127EJW7	tRNA (guanine-N(1)-)-methyltransferase	2.5228	0.000202	Up
A0AAV3BP49	Potassium uptake protein	1.1426	0.000342	Up
A0A6G4ZBX6	Phosphate acyltransferase	2.8481	0.000352	Up
A0A2X2Y5W9	Pyridine nucleotide-disulfide oxidoreductase	1.3727	0.000379	Up
A0A0H2YS72	Glycosyl hydrolase, family 25	−1.8055	0.000416	Down
A0A133MWJ7	DUF1667 domain-containing protein	2.3632	0.000545	Up
A0A0H2YU91	Alpha-*N*-acetylglucosaminidase family protein	−1.5792	0.000632	Down
A0AAE8FUG7	Phage protein	−1.4726	0.000717	Down
A0A127EHE8	Histidine kinase	1.3637	0.00081	Up
A0A0H2YP35	GTP diphosphokinase	1.2855	0.000987	Up
A0AAP6MC58	Beta-ketoacyl-[acyl-carrier-protein] synthase III	1.8706	0.001074	Up
A0AAV3FA78	ATP synthase subunit b	−1.2141	0.00146	Down

^*a*^ Statistically significant proteins were characterized by *P* < 0.05 and log₂ FC < −1 or >1.

^*b*^ FC, fold change (treated/control group).

^*c*^ Statistical analysis was performed with two-tailed Student’s *t*-test from three independent experiments.

**TABLE 3:** Top 20 significantly abundant whole-cell lysate proteins^*a*^ ranked by *P* value

Accession	Description	log_2_FC^*b*^	*P* value^*c*^	Note
A0A0H2YS43	von Willebrand factor type A/Cna B-type domain protein	1.9164	0.000066	Up
A0AAP4ED11	Sirohydrochlorin cobaltochelatase	1.5377	0.000085	Up
A0A127EFP9	YjbQ family protein	1.5751	0.000195	Up
A0A133NCC4	UDP-N-acetylglucosamine 1-carboxyvinyltransferase	1.1524	0.00047	Up
A0A0H2YSC8	Cell wall surface anchor family protein	2.6680	0.000967	Up
A0A133MV71	Extracellular protein	1.0713	0.000989	Up
A0A0H2YPU6	Phage major capsid protein, HK97 family	−2.4989	0.001011	Down
A0A0H2YQ24	Putative dipeptidase	1.0009	0.001366	Up
A0A133NEQ4	Uncharacterized protein	1.3510	0.001544	Up
A0A133NE23	Uncharacterized protein	−1.4949	0.001915	Down
A0A133N6V6	BMC domain protein	1.0457	0.001952	Up
A0A127EJY8	Ribonuclease HII	1.0572	0.002162	Up
A0A0H2YU14	RecT family protein	1.5658	0.002367	Up
A0A173ZPP6	DUF1836 domain-containing protein	1.1667	0.002427	Up
A0A133NEW5	PTS system fructose IIA component	1.0362	0.002655	Up
A0AAE8K7B9	DUF861 domain-containing protein	1.2476	0.002739	Up
A0A2X2YE58	GTP-binding protein	2.4402	0.003345	Up
A0AAP7BWW0	Cof-type HAD-IIB family hydrolase	1.9847	0.004152	Up
A0A0H2YU91	Alpha-N-acetylglucosaminidase family protein	−1.7320	0.004223	Down
A0A0H2YQU5	Ribosome biogenesis GTPase A	1.8544	0.004457	Up

^*a*^ Statistically significant proteins were characterized by *P* < 0.05 and log₂ FC < −1 or >1.

^*b*^ FC, fold change (treated/control group).

^*c*^ Statistical analysis was performed with two-tailed Student’s *t*-test from three independent experiments.

Across all fractions, 225 proteins were significantly altered (*P* < 0.05; log_2_ fold change < –1 or >1) (Fig. 3C). All WCL, surface-associated, and secreted proteins that met the significance criteria, together with their corresponding FDR-adjusted *P* values (*q*-values), are listed in Tables S1 to S3. To highlight the most affected proteins, the top 20 significantly altered proteins from each fraction were presented in Tables 1 to 3, together with the corresponding volcano plots visualizing their differential abundance. Consistent with the PCA results, most changes were observed in the secreted and surface-associated proteins. Of all secreted proteins (Table 1), levels of multiple proteins involved in cell wall surface organization and stress adaptation (e.g., RNase adapter RapZ, 3-oxoacyl-[acyl-carrier-protein] reductase, actin-like protein MreB, and several cell wall surface anchor proteins), proteins involved in the regulation of cell growth (e.g., inosine-5′-monophosphate dehydrogenase), and cytosolic enzymes/proteins (e.g., L-lactate dehydrogenase and chaperone protein ClpB) were markedly elevated in taxifolin-treated cells. The increased abundance of these normally surface-associated or cytosolic proteins in the secreted fraction or supernatant suggests altered protein localization or increased release of surface-associated proteins under stress conditions rather than overt cell lysis (34). Von Willebrand factor type A/Cna B-type domain proteins serve as tip adhesins of pilin and are involved in the adhesion of *C. perfringens* to collagen (32). Their increased concentrations in the extracellular region may explain the reduced adhesion and biofilm formation capacity of taxifolin-treated cells. Additionally, levels of proteins linked to redox or metal-induced stress, such as L-lactate dehydrogenase and the iron–sulfur cluster carrier protein (35, 36), were elevated, indicating activation of oxidative and metal stress response pathways rather than mere suppression of bacterial growth. In the surface-associated fraction (Table 2), the levels of proteins related to cell wall maintenance and environmental sensing, including glycosyl transferases, histidine kinases, and phosphate acyltransferases, were increased. In the WCL fraction (Table 3), levels of several extracellular or cell wall-associated proteins, such as von Willebrand factor domain proteins and UDP-*N*-acetylglucosamine 1-carboxyvinyltransferase (MurA), were also elevated, suggesting a compensatory mechanism to improve cell wall integrity. These coordinated changes suggest that taxifolin perturbs protein dynamics at the bacterial surface and triggers surface-associated stress responses, consistent with the morphological abnormalities observed via SEM (Fig. 1C).

### Effects of metal ions and chelators on *C. perfringens* growth and colonization-associated phenotypes

Iron deprivation affects cell membrane integrity, increases cellular permeability, and induces cell elongation (37, 38), as observed in this study. Polyphenolic compounds, such as taxifolin, chelate transition metals. Previous studies reported that taxifolin forms coordinate complexes with iron ions (39–42). Based on this metal-binding property, we hypothesized that growth inhibition of *C. perfringens* by taxifolin is mediated by the sequestration of essential metal ions, particularly iron.

**Fig 4:**
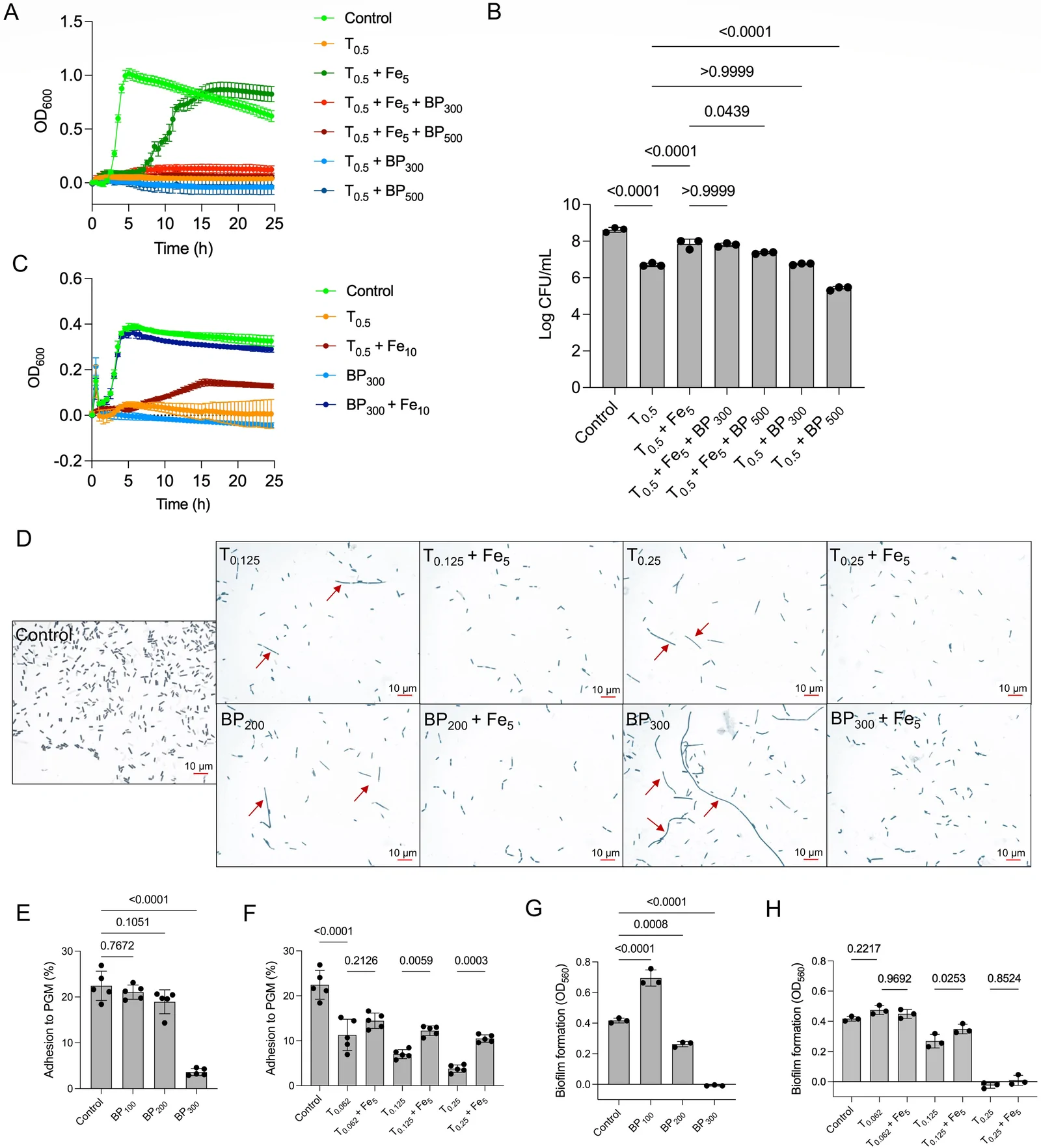
Effects of metal ions and chelators on *C. perfringens* growth, cell morphology, mucin adhesion, and biofilm formation. (**A and B**) Growth of *C. perfringens* in the modified Gifu anaerobic medium (mGAM) containing 0.5 mg/mL taxifolin (T_0.5_) with or without Fe^2+^ supplementation (5 µg/mL; Fe_5_). (**A**) Growth curves monitored by OD_600_. (**B**) Viable cell counts (log CFU/mL) after 24-h incubation with Fe^2+^ supplementation or the iron chelator 2,2′-bipyridyl (BP; 300 µM [BP_300_] or 500 µM [BP_500_]). (**C**) Growth of *C. perfringens* in trypticase soy broth (TSB) supplemented with T_0.5_, BP_300_, and/or Fe^2+^ (10 µg/mL; Fe_10_) monitored by OD_600_. Data are presented as the mean ± SD of three independent experiments. (**D**) Representative light microscopy images of *C. perfringens* cultured in mGAM containing taxifolin or BP, with or without Fe^2+^, at 1,000× magnification. (**E and F**) Mucin adhesion of *C. perfringens*, expressed as the percentage of surface area covered by adherent cells. (**E**) BP-treated groups. (**F**) Taxifolin-treated groups, with or without Fe^2+^ (Fe_5_). Each dot represents a value quantified from an individual microscopic image acquired from a distinct well. Bars represent the mean ± SD. (**G and H**) Biofilm formation of *C. perfringens* on collagen type I. (**G**) BP-treated groups. (**H**) Taxifolin-treated groups, with or without Fe^2+^ (Fe_5_). (**B, E–H**) Statistical significance was determined via one-way ANOVA, followed by Tukey’s post-hoc test (*P* < 0.05). BP_100_, 100 µM 2,2′-bipyridyl; BP_200_, 200 µM 2,2′-bipyridyl; BP_300_, 300 µM 2,2′-bipyridyl; BP_500_, 500 µM 2,2′-bipyridyl; PGM, porcine gastric mucin; T_0.062_, 0.062 mg/mL taxifolin; T_0.125_, 0.125 mg/mL taxifolin; T_0.25_, 0.25 mg/mL taxifolin; T_0.5_, 0.5 mg/mL taxifolin.

Fe^2+^ has been shown to significantly enhance *C. perfringens* growth in demetalled medium (27). As shown in Fig. 4A, supplementation with Fe^2+^ (5 µg/mL, hereafter Fe_5_) markedly improved the growth of bacteria treated with 0.5 mg/mL taxifolin (inhibitory concentration) in mGAM. Meanwhile, the addition of BP, an iron-specific chelator, at 300 and 500 µM (BP_300_ and BP_500_, respectively) abolished this restorative effect. Higher Fe^2+^ concentrations (10 µg/mL) also exerted similar effects on cells (data not shown). Consistent with these observations, Fe_5_ supplementation significantly increased, whereas BP_500_ addition significantly reduced the viable counts (Fig. 4B). To further validate this mechanism under nutrient-limited conditions, assays were performed in TSB medium (Fig. 4C). Notably, growth was completely inhibited by BP and restored by 10 µg/mL Fe^2+^ (Fe_10_) supplementation, confirming the essential role of iron. Similarly, taxifolin (0.5 mg/mL) inhibited bacterial growth, but Fe^2+^ supplementation partially restored it by approximately 50%.

To investigate whether the inhibitory effect of taxifolin could also be attributed to the chelation of metal ions other than iron, similar growth rescue experiments were performed with Ca^2+^, Mn^2+^, Zn^2+^, and Mg^2+^. In contrast to Fe^2+^, supplementation with these metal ions (5 µg/mL) failed to restore the growth of *C. perfringens* in the presence of an inhibitory concentration of taxifolin (Fig. S7A). Growth remained suppressed when higher concentrations (up to 20 µg/mL) of these ions were tested (data not shown), supporting a specific role for iron in the observed growth recovery.

To assess whether this response was conserved in other susceptible bacteria, the iron supplementation experiments were also performed with *C. difficile*, which also exhibits a similar sensitivity to iron availability. At 1 mg/mL taxifolin, supplementation with 5 µg/mL Fe^2+^, but not higher concentrations (10 µg/mL), markedly improved the growth of *C. difficile* (Fig. S7B). Similar Fe^2+^-dependent growth recovery was also observed in other *C. perfringens* strains, although the magnitude of rescue was less pronounced than that observed for *C. perfringens* JCM 1290^T^ (Fig. S7C).

Microscopic observation revealed marked elongation of *C. perfringens* cells under iron-limited conditions, induced by either taxifolin or BP treatment (Fig. 4D). Taxifolin at 0.125 mg/mL and 0.25 mg/mL caused noticeable cell elongation compared with the control. This effect was even more pronounced in BP-treated cells, where cells appeared thread-like when treated with BP_300_. Notably, Fe^2+^ supplementation restored normal cell morphology across all taxifolin and BP treatments, comparable to that of the control.

To determine whether the effect of Fe^2+^ extends to colonization-related processes, we examined the mucin adhesion and biofilm formation of *C. perfringens* under iron-limited (taxifolin and BP) and -repleted conditions. Similar to taxifolin, BP significantly reduced mucin adhesion and biofilm formation of *C. perfringens*, with effects observed from 300 µM and 200 µM, respectively (Fig. 4E and G). Meanwhile, Fe^2+^ supplementation increased both mucin adhesion and biofilm formation compared with the corresponding taxifolin-treated groups (≥0.125 mg/mL) (Fig. 4F and H). These findings support a role for iron in both growth and colonization-related phenotypes of *C. perfringens*. Further experiments confirmed that taxifolin significantly reduced free iron levels in the medium, albeit to a lesser extent than BP (Fig. S8). Collectively, these results suggest that iron chelation is an important mechanism contributing to the antibacterial activity of taxifolin against *C. perfringens*.

## DISCUSSION

In this study, we found that taxifolin exhibited the strongest inhibitory activity against *C. perfringens* among all the bacterial species tested. Our data suggest that this enhanced susceptibility is associated with iron limitation-related physiological stress. Our findings provide mechanistic insights into the inhibition of *C. perfringens* by dietary flavonoids.

Taxifolin exerted limited effect on the growth of most representative commensal bacteria tested in this study (Fig. S2). However, modest inhibition was observed for certain *Clostridium* species, including *C. butyricum* and *C. sporogenes*, with detectable inhibitory effects also observed for *C. difficile*. Together with the pronounced sensitivity of *C. perfringens*, these observations suggest that taxifolin acts in a species- and strain-dependent manner, rather than uniformly targeting broad bacterial groups. Taxifolin has been primarily investigated for its antioxidant and cytoprotective properties. Previous studies have reported the attenuation of virulence-associated pathways in certain pathogens, such as methicillin-resistant *S. aureus*, through mechanisms distinct from those identified in this study, including sortase A inhibition (22). These reports further underscore that the antibacterial effects of taxifolin are highly context- and species-dependent.

Differences in the bacterial cell surface architecture may have partially contributed to the observed susceptibility patterns. The reduced sensitivity of many gram-negative bacteria to taxifolin may reflect the presence of an outer membrane that limits the entry of hydrophobic flavonoids (43–46). Although this structural distinction alone cannot fully explain the enhanced susceptibility of *C. perfringens*, it possibly contributed to the lower susceptibility of gram-negative species. Previous *in vivo* studies have reported the microbiota-modulating effects of taxifolin, including the enrichment or restoration of specific *Clostridium* populations, partial normalization of disease-associated dysbiosis, and correction of altered Firmicutes/Bacteroidetes ratios in aged or colitic mouse models (14, 17, 19, 47, 48). Consistent with these observations, the limited impacts of taxifolin on most commensal bacteria in this study suggest that its inhibitory effects are not broadly disruptive to the gut microbiota.

Adhesion to host tissues and biofilm formation are important virulence-associated traits that enable foodborne pathogens to persist in the intestinal environment and tolerate environmental stress (49). Although toxin production is the primary virulence determinant of *C. perfringens*, increasing evidence indicates that adhesion and biofilm formation substantially contribute to its pathogenic persistence and disease severity (11, 32, 50). In this study, taxifolin significantly reduced *C. perfringens* adhesion and biofilm formation at subinhibitory concentrations (Fig. 2B and C), suggesting impairment of colonization-associated phenotypes. Bacterial adherence is linked to prolonged and severe disease manifestations in certain *C. perfringens* infections, including non-foodborne gastrointestinal diseases caused by type F strains in humans (33) and enteritis in poultry (51).

Consistent with our *in vitro* findings, taxifolin administration reduced *C. perfringens* colonization during the early phase of infection in the antibiotic-pretreated mouse model (Fig. 2E). This reduction was most pronounced shortly after challenge and gradually diminished over time, suggesting that taxifolin limits early intestinal expansion or persistence rather than completely eradicating the pathogen. The *C. perfringens* JCM1290^T^ strain used in this study is a bovine-derived type strain and is classified as toxinotype A. Toxinotype A strains typically produce phospholipase C (alpha toxin) and may also produce several hemolytic and other cytotoxic factors. Although *C. perfringens* is an important causative agent of gas gangrene and enteric diseases, JCM 1290ᵀ induces overt intestinal pathology in mice only under intensive experimental conditions, such as high-dose inoculation or repeated daily challenge (52). This strain was selected as it showed the highest sensitivity to taxifolin in the initial screening and is a type strain, as the primary focus of this study was bacterial intestinal colonization rather than toxin-mediated pathology. Accordingly, the mice maintained relatively stable body weights throughout the experiment. Nevertheless, elevated intestinal burdens of *C. perfringens* have been associated with increased tissue damage, inflammation, and barrier dysfunction in murine models (33, 53). Therefore, the observed reduction in fecal *C. perfringens* loads in taxifolin-treated groups reflects the effect on bacterial colonization dynamics rather than the attenuation of disease severity, although it is consistent with reduced pathogenic potential. Following taxifolin administration, a pronounced reduction in *C. perfringens* colonization was observed on days 1 and 3 post-infection. Notably, a gradual decrease in bacterial load was also observed in the control group on days 5 and 7 post-infection. While host immune responses are possibly involved in the control of *C. perfringens* colonization throughout infection, as previously reported (54), the present data suggest that taxifolin may facilitate this process, without directly addressing the underlying immune mechanisms. Similar protective effects have been reported for flavonoids or flavonoid-rich diets in *C. perfringens*-associated intestinal disease models (55–57). Our study extends these observations by demonstrating that taxifolin reduces early intestinal colonization in a *C. perfringens*-challenged mouse model. However, the present study did not directly assess toxin production, host inflammatory responses, or changes in gut microbiota composition. Therefore, the possibility that host- or microbiota-mediated mechanisms contributed to the reduced colonization cannot be excluded.

To gain insights into the mechanism by which taxifolin perturbs *C. perfringens* physiology, we performed comparative proteomic analyses to identify the proteins responsive to taxifolin exposure at 0.125 mg/mL. Two related response patterns emerged that were consistent with the observed physiological and morphological phenotypes. A prominent feature of proteomic data was the disruption of iron homeostasis. Proteins typically induced under iron-limiting conditions, including iron–sulfur cluster carrier proteins and other redox-associated enzymes, were consistently enriched in the extracellular fraction, suggesting reduced availability of intracellular iron. This interpretation was supported by metal supplementation experiments; Fe^2+^ supplementation markedly restored taxifolin-inhibited growth, whereas the iron chelator BP abolished this rescue effect. This trend extends to colonization-related phenotypes, as mucin adhesion and biofilm formation of *C. perfringens* were both enhanced by Fe^2+^ supplementation compared with the corresponding taxifolin-treated groups. These findings are consistent with the known iron-chelating capacity of taxifolin mediated by its catechol moiety (16, 40). Because *C. perfringens* relies heavily on iron-dependent enzymes and Fe–S cluster proteins to sustain its anaerobic metabolism and redox balance (28, 58), even modest reductions in accessible iron may substantially impair its growth and physiological functions. Therefore, iron availability is an important regulator of bacterial adhesion and biofilm formation in various species. Iron depletion reduces biofilm formation in *Pseudomonas aeruginosa* (59), *S. aureus* (60), and *S. epidermidis* (61), as *S. aureus* requires iron for primary attachment and synthesis of polysaccharide intercellular adhesin. The iron-dependent restoration of growth, mucin adhesion, and biofilm formation of *C. perfringens* suggests that iron limitation contributes to taxifolin-mediated suppression of colonization.

Along with the disruption of iron homeostasis, proteins involved in the maintenance of the gram-positive cell surface and associated stress responses were among the most prominently altered. Several proteins typically localized to the cell surface or cytosol, including MreB, cell wall surface anchor proteins, and L-lactate dehydrogenase, were detected at elevated levels in the extracellular fraction, suggesting increased leakage or altered protein localization. Previous studies have reported that flavonoids perturb bacterial cell membrane integrity, as evidenced by increased membrane fluidity and conductivity and enhanced release of intracellular components (34, 62, 63). Consistently, levels of proteins involved in cell wall biosynthesis, surface organization, environmental sensing, and adhesion, including MurA, multiple glycosyltransferases involved in peptidoglycan synthesis, and von Willebrand factor type A/Cna B-type domain-containing proteins, were elevated in the surface-associated and whole-cell fractions in this study. Similar induction of genes encoding cell wall biosynthesis and modification enzymes (e.g., *murG*, *murD*, and *murF*) has been reported in *C. difficile* under iron-limited conditions (64). Although overt cell surface damage was not clearly observed via electron microscopy under the tested conditions, these proteomic signatures suggest that taxifolin perturbs cell surface homeostasis, inducing compensatory upregulation of cell wall-associated proteins rather than causing extensive membrane disruption.

Consistent with an iron-limited physiological state, taxifolin induced pronounced morphological alterations, most notably marked cell elongation with occasional evidence of incomplete septation (Fig. 1C and 4D). The same trend was also observed in cells treated with BP, a strong iron chelator. Notably, Fe^2+^ supplementation partially restored cell morphology. Similar elongation phenotypes have been reported in *C. perfringens* under Mg^2+^ or K^+^ deficiency (27) and in *C. welchii* and *E. coli* under iron-limited conditions (65, 66). In *E. coli*, iron starvation-induced sRNA *RyhB* downregulates the cell division protein ZapB, resulting in impaired septation and elongated cells containing additional chromosomes (66). Cell elongation is also observed in *C. cellulovorans* under butanol stress (67) and represents a general adaptive response that reduces the surface-to-volume ratio and limits the entry of toxic compounds (68). Together, the proteomic alterations and elongation phenotypes observed by microscopy suggest that taxifolin disrupts normal septum formation and cell surface stability primarily via stress-associated physiological adaptation rather than direct bacteriolytic damage.

Collectively, our findings support a model in which the inhibitory effects of taxifolin on *C. perfringens* are associated with physiological stress responses involving iron homeostasis, cell surface-associated processes, and cell division, rather than direct bactericidal activity. This multifactorial mode of action provides a plausible explanation for the observed suppression of bacterial growth, adhesion, and biofilm formation. In contrast to conventional bactericidal antibiotics, taxifolin predominantly acts in a bacteriostatic manner, reducing excessive damage to bacterial cell integrity while effectively limiting pathogenic behaviors. Notably, taxifolin at concentrations ≤0.125 mg/mL did not show detectable cytotoxicity in intestinal epithelial cells (Fig. S9), indicating that biologically effective concentrations are well tolerated by host cells. Moreover, taxifolin exerted minimal effects on most representative commensal bacteria tested in this study, particularly at the subinhibitory concentrations against *C. perfringens*. Although the impact of taxifolin on the gut microbiota was not directly evaluated *in vivo*, these findings suggest that taxifolin may function as a dietary-derived modulator of enteric pathogens with limited effects on non-target bacteria.

Because subinhibitory concentrations of taxifolin were sufficient to significantly reduce mucin adhesion and biofilm formation, most subsequent experiments were conducted at these concentrations. The absence of overt cell surface damage in the SEM observations may therefore reflect adaptive physiological stress rather than gross structural disruption. Notably, only Fe^2+^, and not Mg^2+^, Mn^2+^, Zn^2+^, or Ca^2+^, partially restored bacterial growth, supporting a specific role for iron availability in the inhibitory effects of taxifolin on *C. perfringens*. However, the incomplete rescue by Fe^2+^ suggests that additional mechanisms may also be involved. Because intracellular iron levels were not directly measured, the extent of intracellular iron deprivation remains to be determined.

Consistent with its proposed role as a dietary component rather than a therapeutic antimicrobial, taxifolin conferred only modest suppression of infection in the murine model, supporting its potential use as a preventive dietary agent to attenuate bacterial virulence and fitness rather than eliminate the pathogen. In the present study, the impact of taxifolin on *C. perfringens* infection was evaluated primarily through fecal bacterial enumeration. Consequently, additional studies incorporating histopathological analyses, toxin quantification, inflammatory markers, and microbiome profiling will be necessary to further clarify the *in vivo* consequences and the mechanisms underlying taxifolin-mediated protection.

It is also noteworthy that susceptibility to taxifolin varied among the *C. perfringens* strains tested, indicating that taxifolin susceptibility should be considered a strain-specific trait rather than a uniform characteristic of the species. Clostridia rely on iron-containing enzymes, iron–sulfur proteins, and ferredoxin-dependent pathways for anaerobic metabolism, and iron limitation has been shown to induce marked metabolic reprogramming in *C. difficile* (64, 69). Iron homeostasis also contributes to bacterial growth, stress adaptation, and virulence regulation through Fur-dependent regulatory systems (70). *C. perfringens* has an open pangenome with substantial variation in genes involved in iron acquisition and other physiological functions (71), which may contribute to the observed strain-dependent differences. Further studies will be required to determine the genetic and physiological basis of this variation in individual strains.

## Data Availability

All proteomic data sets, including protein identifications and quantitative analyses, are available in the Supplemental Materials accompanying this manuscript.

## SUPPLEMENTAL MATERIAL

Supplemental figure spectrum.01301-26-s0001.pdf Fig. S1 to S9.

Supplemental tables spectrum.01301-26-s0002.xlsx Tables S1 to S3.

ASM does not own the copyrights to Supplemental Material that may be linked to, or accessed through, an article. The authors have granted ASM a non-exclusive, world-wide license to publish the Supplemental Material files. Please contact the corresponding author directly for reuse.
