# Supplementary material for: Taxifolin impairs colonization of Clostridium perfringens in association with iron limitation-related physiological stress
Source: Microbiol Spectr. 2026 Aug 21;14(10):e01301-26. doi: 10.1128/spectrum.01301-26 (PMC13689440; doi:10.1128/spectrum.01301-26)
Supplement: Supplementary file 1 [file spectrum.01301-26-s0001.pdf]

Supplementary data

Taxifolin impairs colonization of *Clostridium perfringens* in association with iron limitation-related physiological stress

Fadhilatunnur et al.

Supplementary Figures S1–S9

Fig. S1. MIC_{70} of taxifolin against pathogenic bacteria.

Fig. S2. Effect of taxifolin on the growth of representative commensal gut bacteria.

Fig. S3. Strain-dependent growth inhibition of *C. perfringens* by taxifolin.

Fig. S4. Quantification of cell elongation in *C. perfringens* treated with taxifolin.

Fig. S5. Body weight changes of mice during *C. perfringens* challenge with taxifolin treatment.

Fig. S6. Protein fractionation profiles and principal component analysis of taxifolin-treated *C. perfringens*.

Fig. S7. Effect of divalent metal ions on the growth of *C. perfringens* and *C. difficile* under taxifolin treatment.

Fig. S8. Cell-free assessment of iron-chelating capacity of taxifolin and 2,2′-bipyridyl.

Fig. S9. Cytotoxicity assessment of taxifolin in porcine intestinal epithelial (INV-1–3) cells.

Supplementary Tables S1–S3 (Tables S1–S3 are provided as separate Excel files.)

Table S1. Differentially expressed proteins identified in whole-cell lysate (WCL) fractions of *C. perfringens* following taxifolin treatment.

Table S2. Differentially expressed proteins identified in surface-associated fractions of *C. perfringens* following taxifolin treatment.

Table S3. Differentially expressed proteins identified in secreted/culture supernatant fractions of *C. perfringens* following taxifolin treatment.

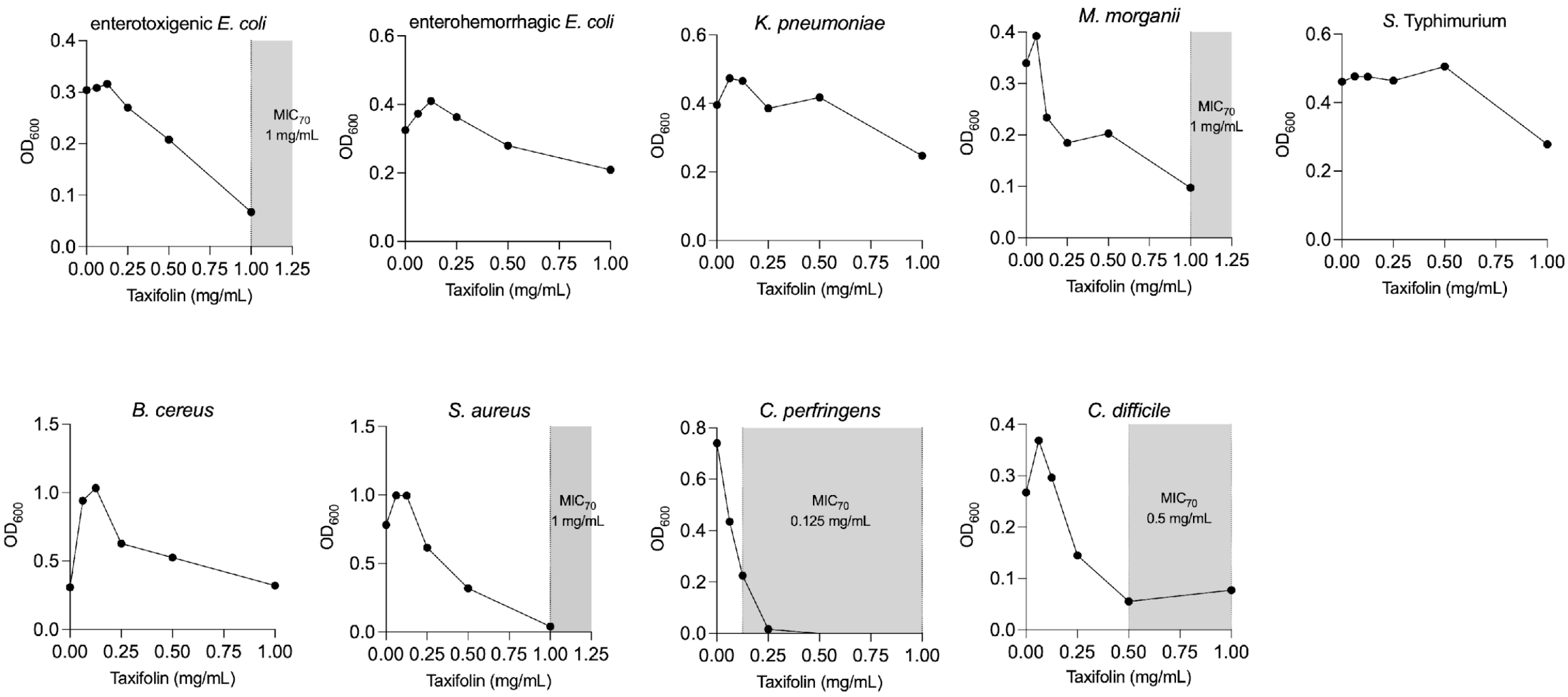

Fig. S1. MIC70 of taxifolin against pathogenic bacteria.

MIC_{70} values of taxifolin against pathogenic bacterial species. MIC_{70} was defined as the concentration of taxifolin that resulted in ≥70% growth inhibition at the time point when the untreated control culture reached maximal OD_{600}. Growth was monitored by OD_{600}, and values represent the mean ± SD of three independent biological replicates.

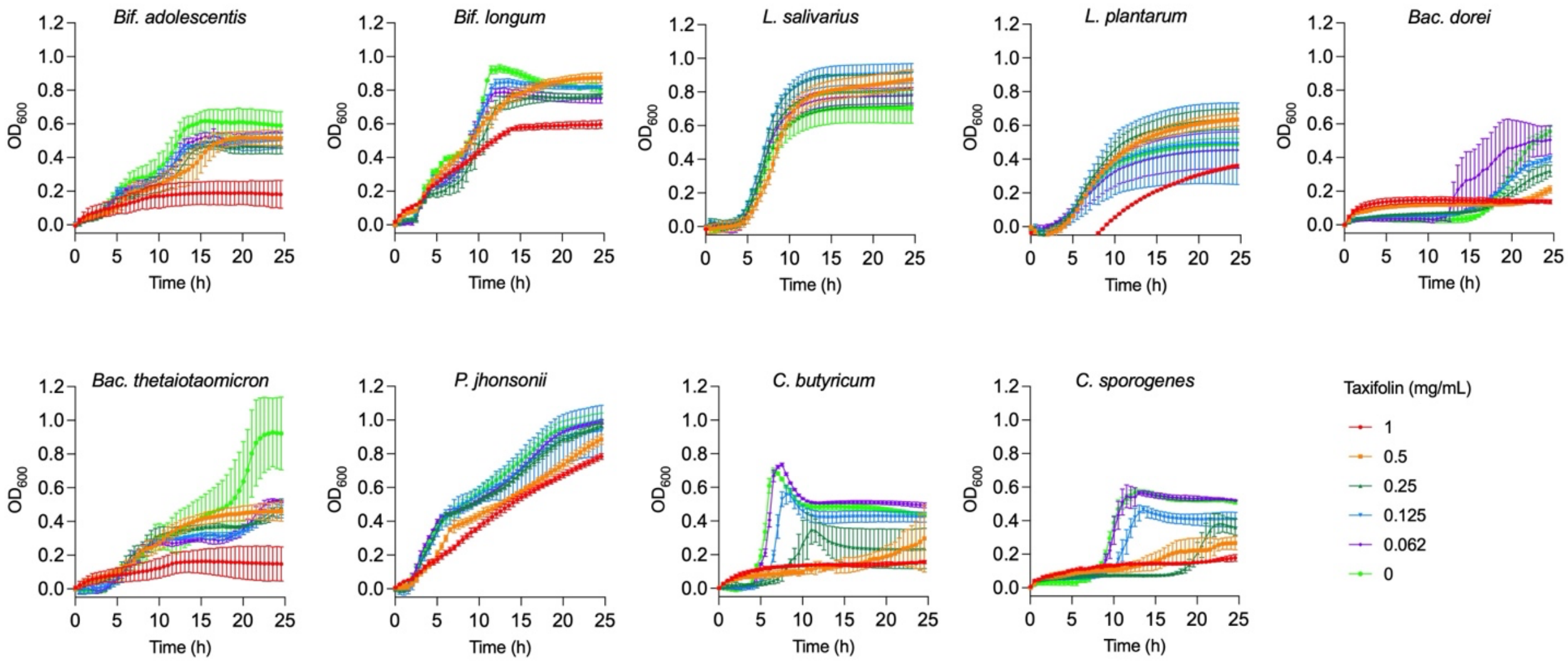

Fig. S2. Effect of taxifolin on the growth of representative commensal gut bacteria.

Growth curves of representative commensal gut bacteria cultured in the presence of increasing concentrations of taxifolin. Bacterial growth was monitored by OD_{600}. Data represent the mean ± SD of three independent biological replicates.

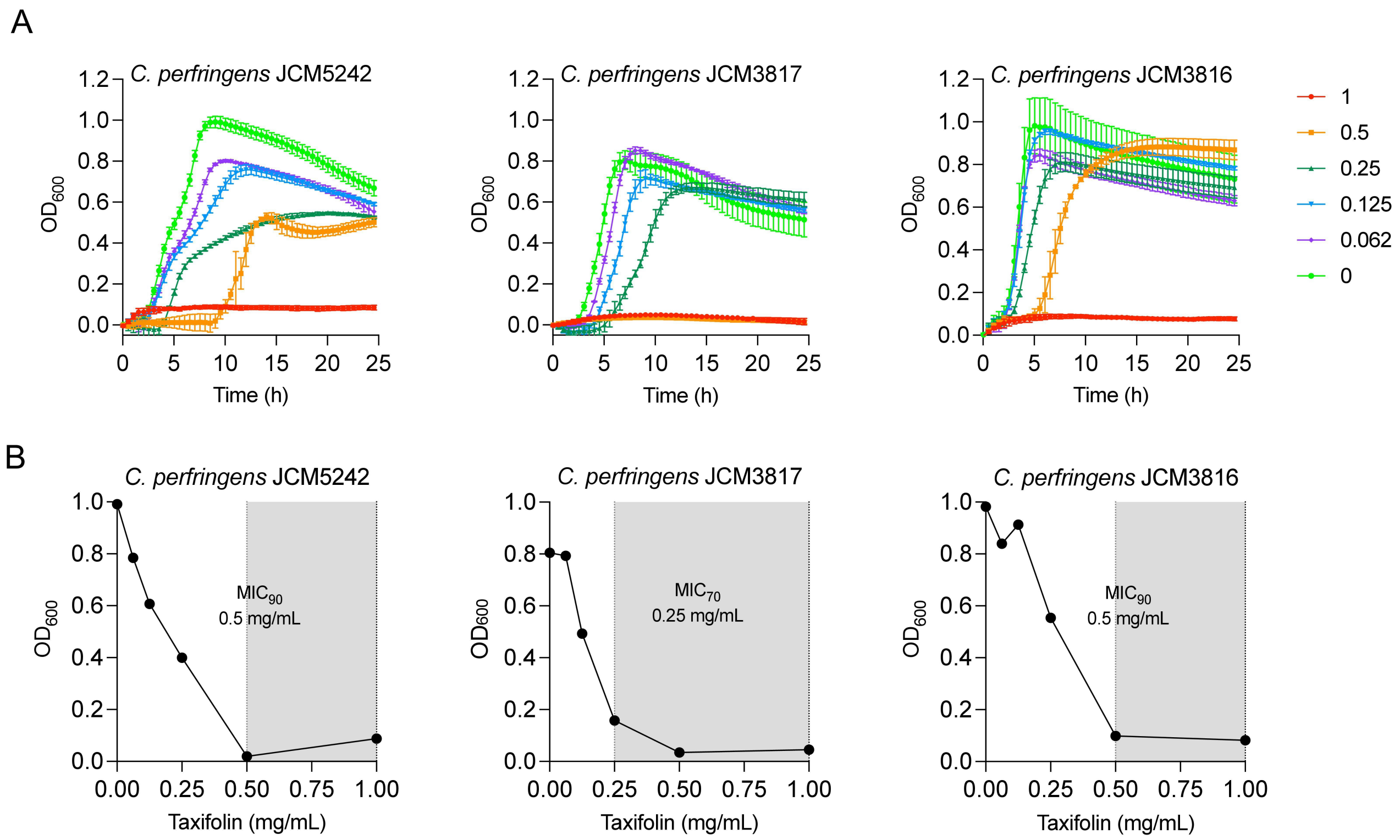

Fig. S3. Strain-dependent growth inhibition of *C. perfringens* by taxifolin.

(A) Growth curves of different *C. perfringens* strains cultured in the presence of increasing concentrations of taxifolin and **(B)** the corresponding MICs. Growth was monitored by OD_{600}. Data represent the mean ± SD of three independent biological replicates. The MIC was defined as the concentration of taxifolin that resulted in the indicated growth inhibition at the time point when the untreated control culture reached maximal OD_{600}.

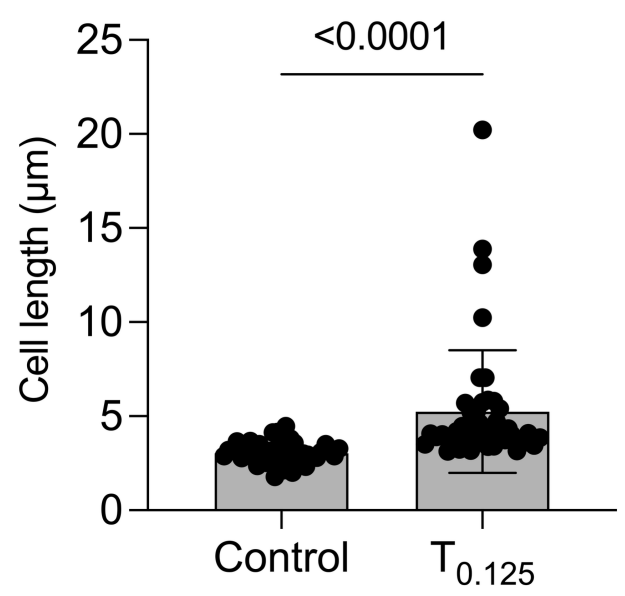

Fig. S4. Quantification of cell elongation in *C. perfringens* treated with taxifolin.

Average cell length of *C. perfringens* cultured with taxifolin (0.125 mg/mL). Individual data points represent measurements from single bacterial cells (*n* = 44 cells per condition). Bars indicate the mean ± SD. Statistical significance was determined using a two-tailed Student's *t* test.

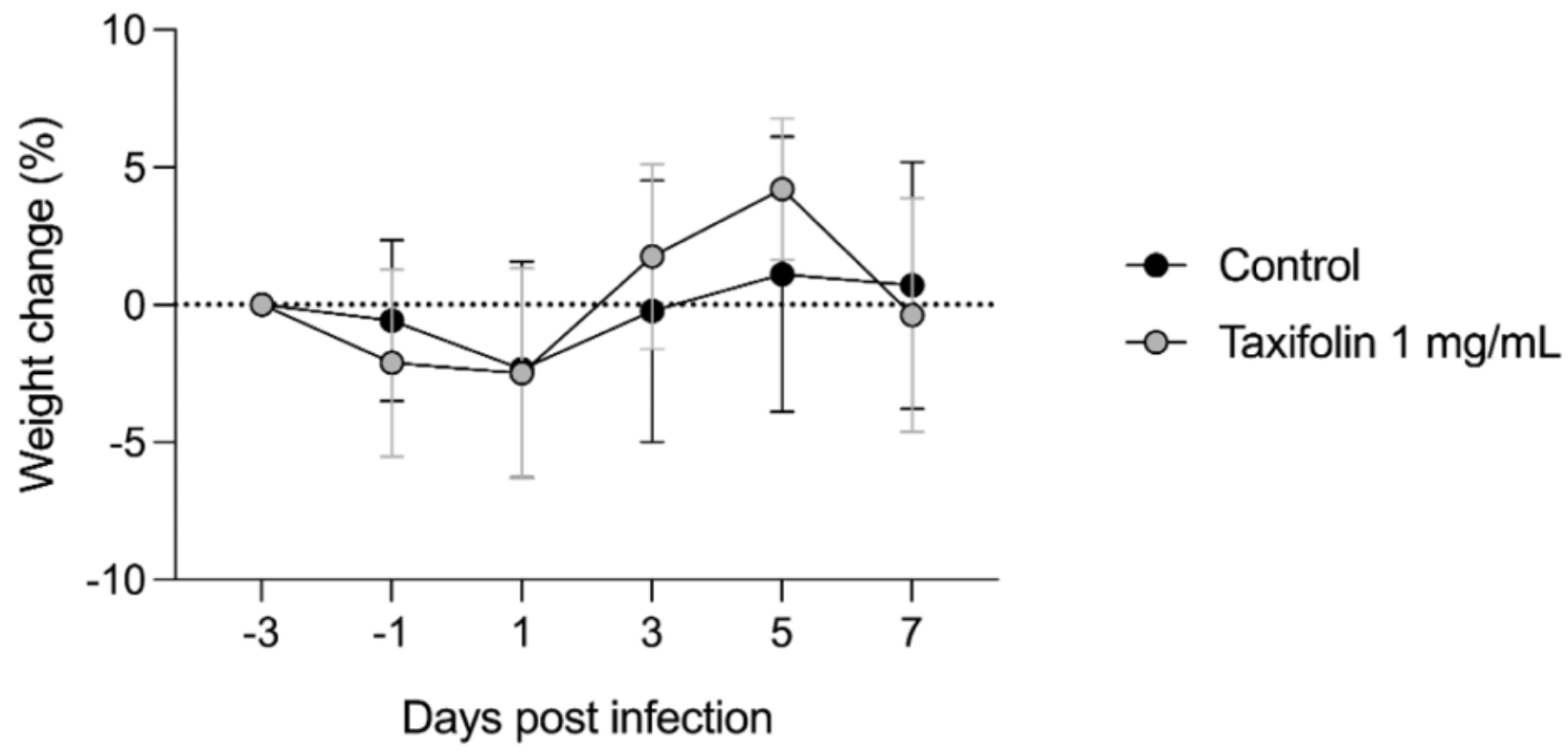

Fig. S5. Body weight changes of mice during *C. perfringens* challenge with taxifolin treatment. Relative body weight changes (%) of mice during the *C. perfringens* challenge experiment. Data represent the mean ± SD from 12 mice/group. Statistical significance was determined using the Mann–Whitney *U* test.

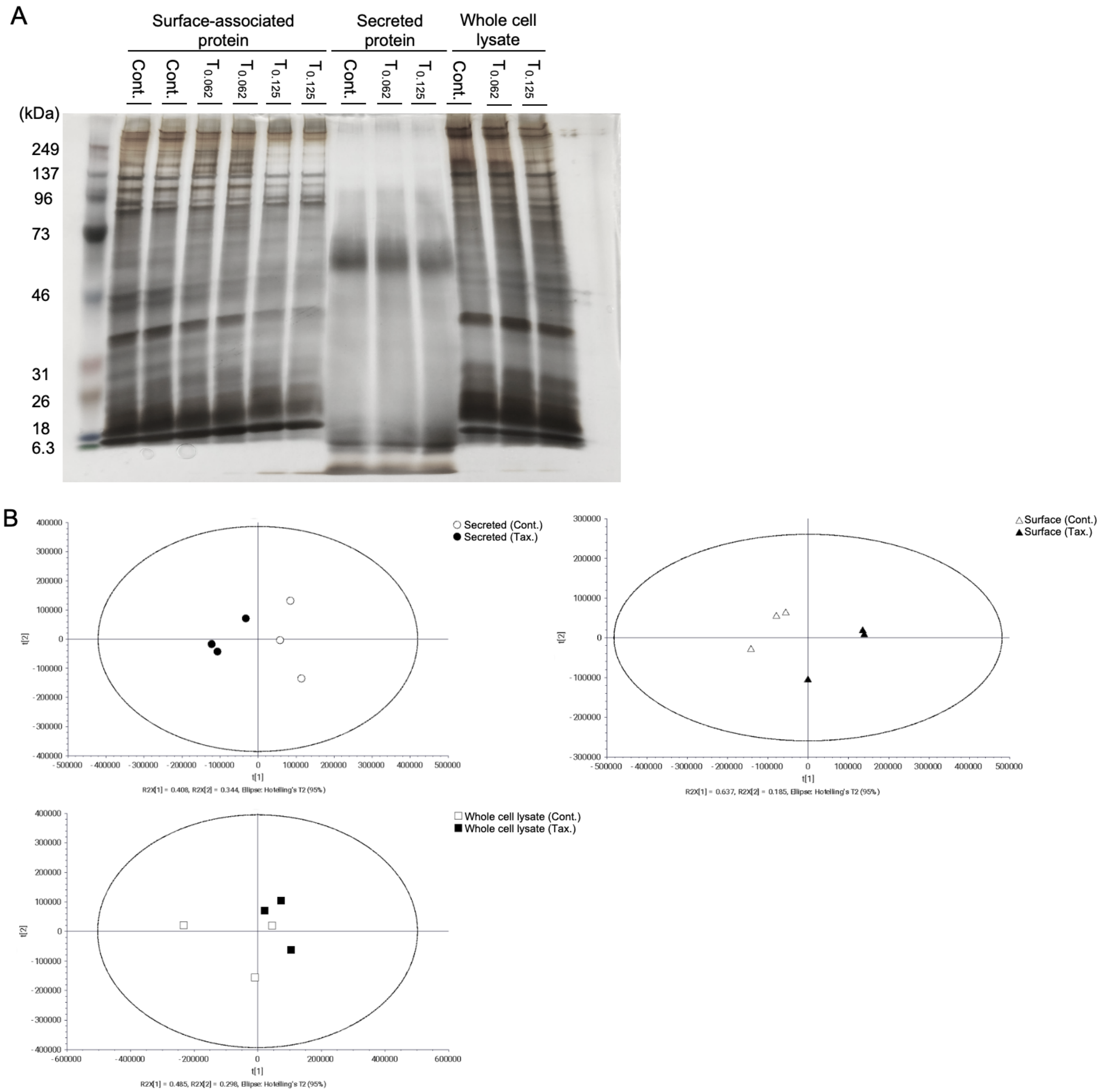

Fig. S6. Protein fractionation profiles and principal component analysis of taxifolin-treated *C. perfringens*.

(A) SDS–PAGE profiles of surface-associated, secreted, and whole-cell lysate proteins from *C. perfringens* treated with taxifolin. **(B)** Principal component analysis (PCA) of proteomic datasets derived from surface-associated, secreted, and whole-cell lysate proteins.

Abbreviations: Cont., control; $T_{0.062}$, 0.062 mg/mL taxifolin; $T_{0.125}$, 0.125 mg/mL taxifolin; $T_{0.25}$, 0.25 mg/mL taxifolin.

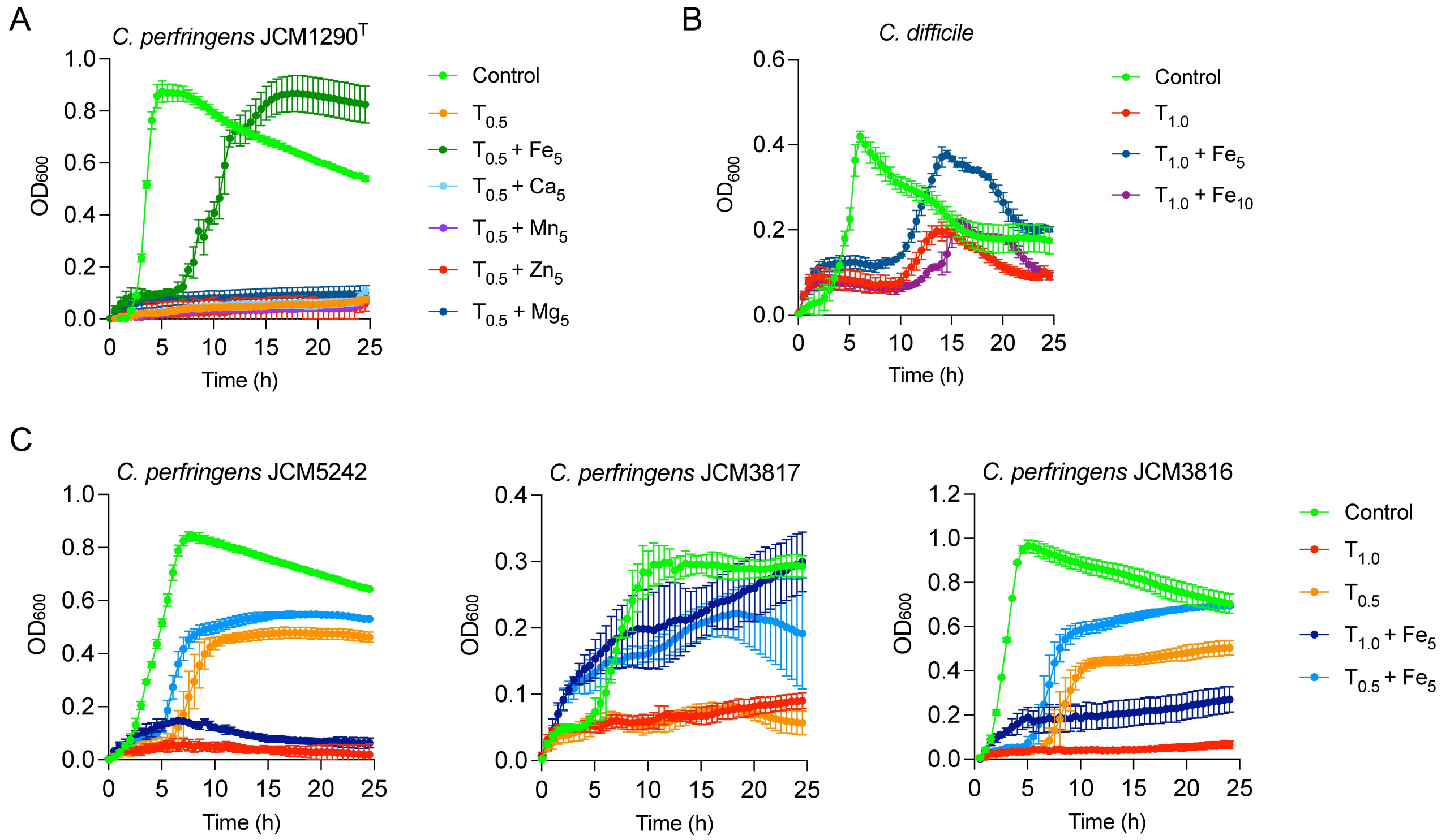

Fig. S7. Effect of divalent metal ions on the growth of *C. perfringens* and *C. difficile* under taxifolin treatment.

(A) Growth of *C. perfringens* cultured in modified Gifu anaerobic medium (mGAM) containing 0.5 mg/mL taxifolin ($T_{0.5}$) and supplemented with 5 µg/mL Fe^{2+}, Ca^{2+}, Mn^{2+}, Zn^{2+}, and Mg^{2+}. **(B)** Growth of *C. difficile* cultured in Gifu anaerobic medium (GAM) containing 1 mg/mL taxifolin ($T_{1.0}$) and supplemented with 5 or 10 µg/mL Fe^{2+} (Fe_5 and Fe_{10} respectively). **(C)** Growth of different *C. perfringens* strains cultured in mGAM containing taxifolin and supplemented with Fe_5. Growth was monitored by OD_{600}. Data represent the mean ± SD of three independent biological replicates.

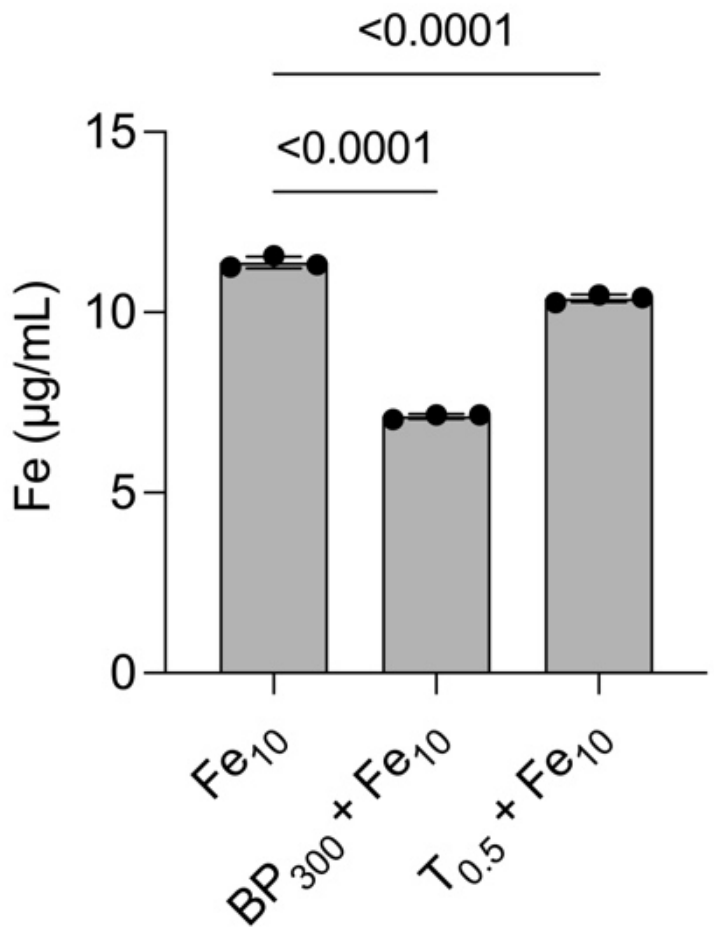

Fig. S8. Cell-free assessment of iron-chelating capacity of taxifolin and 2,2′-bipyridyl.

Quantification of free iron levels in a cell-free system containing Fe^{2+} (10 μg/mL) following the addition of taxifolin (0.5 mg/mL) or 2,2′-bipyridyl (300 μM). Free iron levels are expressed in μg/mL. Data represent the mean ± SD from three independent biological replicates. Statistical significance was determined by one-way ANOVA followed by Dunnett's post hoc test compared with the Fe^{2+} control group.

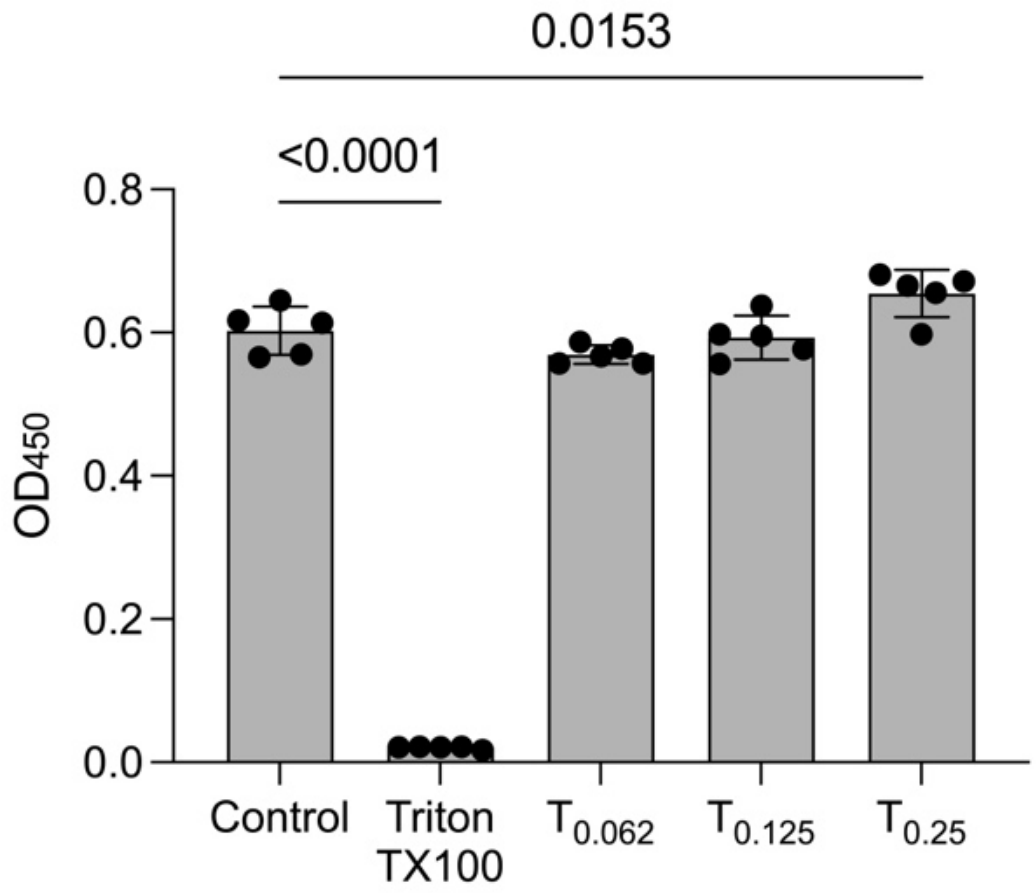

Fig. S9. Cytotoxicity assessment of taxifolin in porcine intestinal epithelial (INV-1–3) cells.

Cytotoxicity of taxifolin toward INV-1–3 cells assessed using a cell counting kit-8 assay. Cells were treated with increasing concentrations of taxifolin, and cell viability was measured after incubation. Bars represent the mean ± SD of five independent experiments, and each dot indicates an individual replicate. Statistical significance was determined by one-way ANOVA followed by Dunnett's post hoc test compared with the untreated control group. Abbreviations: $T_{0.062}$, 0.062 mg/mL taxifolin; $T_{0.125}$, 0.125 mg/mL taxifolin; $T_{0.25}$, 0.25 mg/mL taxifolin.
